# Effect of quinoline based 1,2,3-triazole and its structural analogues on growth and virulence attributes of *Candida albicans*

**DOI:** 10.1371/journal.pone.0175710

**Published:** 2017-04-21

**Authors:** Mohammad Irfan, Shadab Alam, Nikhat Manzoor, Mohammad Abid

**Affiliations:** 1 Medicinal Chemistry Laboratory, Department of Biosciences, Jamia Millia Islamia, New Delhi, India; 2 Medical Mycology Laboratory, Department of Biosciences, Jamia Millia Islamia, New Delhi, India; 3 College of Applied Medical Sciences, Taibah University, Al-Madinah Al-Munawarah, KSA; Yonsei University, REPUBLIC OF KOREA

## Abstract

*Candida albicans*, along with some other non-albicans *Candida* species, is a group of yeast, which causes serious infections in humans that can be both systemic and superficial. Despite the fact that extensive efforts have been put into the discovery of novel antifungal agents, the frequency of these fungal infections has increased drastically worldwide. In our quest for the discovery of novel antifungal compounds, we had previously synthesized and screened quinoline containing 1,2,3-triazole (**3a**) as a potent *Candida* spp inhibitor. In the present study, two structural analogues of **3a** (**3b** and **3c**) have been synthesized to determine the role of quinoline and their anti-*Candida* activities have been evaluated. Preliminary results helped us to determine **3a** and **3b** as lead inhibitors. The IC_50_ values of compound **3a** for *C*. *albicans* ATCC 90028 (standard) and *C*. *albicans* (fluconazole resistant) strains were 0.044 and 2.3 μg/ml, respectively while compound **3b** gave 25.4 and 32.8 μg/ml values for the same strains. Disk diffusion, growth and time kill curve assays showed significant inhibition of *C*. *albicans* in the presence of compounds **3a** and **3b**. Moreover, **3a** showed fungicidal nature while **3b** was fungistatic. Both the test compounds significantly lower the secretion of proteinases and phospholipases. While, **3a** inhibited proteinase secretion in *C*. *albicans* (resistant strain) by 45%, **3b** reduced phospholipase secretion by 68% in *C*. *albicans* ATCC90028 at their respective MIC values. Proton extrusion and intracellular pH measurement studies suggested that both compounds potentially inhibit the activity of H^+^ ATPase, a membrane protein that is crucial for various cell functions. Similarly, 95–97% reduction in ergosterol content was measured in the presence of the test compounds at MIC and MIC/2. The study led to identification of two quinoline based potent inhibitors of *C*. *albicans* for further structural optimization and pharmacological investigation.

## Introduction

Although sincere efforts are being continuously made for discovering new antifungal targets and drugs, the frequency of human fungal infections has increased drastically worldwide, [1–3]. Of particular concern are the ever-increasing incidences of hospital-acquired systemic mycoses caused by *Candida* species responsible for crude mortality rates of up to 50% in the United States alone [4]. Adding to this disease burden, superficial infections of skin and nails in humans are affecting ~25% of the general population worldwide [5]. Use of broad-spectrum antibiotics, suppression of immune response during organ transplantation, immune-suppressive agents during cancer treatment and HIV/AIDS cases have increased the chances of *Candida* spp infections, and hence further aggravating the condition [6]. Among different *Candida* spp, *C*. *albicans* is the major cause of candidiasis and accounts for 80% of the isolates from all forms of human candidiasis [7]. However, the number of infections caused by other non-*albicans Candida* species which includes *C*. *glabrata*, *C*. *tropicalis*, and *C*. *parapsilosis* has also increased significantly [8].

During both superficial and systemic infections, pathogenicity of *Candida* spp relies on a number of virulence factors including morphogenesis and capability to produce hydrolytic enzymes such as proteinases, phospholipases, and lipases. The ability of *C*. *albicans* to switch reversibly between yeast to filamentous or hyphal (pseudo or true, based on condition) form of growth has been well reported as an important virulence attribute [9]. Similarly, hydrolytic enzymes especially proteinases, phospholipases, and lipases help *Candida* spp with adhesion, invasion, host tissue damage and protection from host defense mechanism [10]. Various studies have explained the potential role of these hydrolytic enzymes in the pathogenicity of *Candida* spp [10–13]. In the modern age of drug discovery, the structure and function of potent targets play a very important role in designing better prototypic antimicrobial molecules. H^+^ ATPase, a member of P-type transport ATPase family, has been reported as a potential antifungal target [14–16]. This protein is essentially involved in the physiological functions of *Candida* spp such as maintenance of electrochemical gradient across cell membrane, nutrient uptake, regulation of intracellular pH and cell growth [17]. Plasma membrane H^+^ ATPase is unique to the fungus and is not available as a human protein. Hence this enzyme is crucial to the fungus and maybe explored as a potential antifungal target. Similarly, cytochrome P450-dependent enzyme lanosterol 14 α-demethylase (CYP51) is involved in the biosynthesis of ergosterol in fungi which is an important component of fungal membranes. Lanosterol 14 α-demethylase has already been explored as a potential drug target for azole based antifungal agents [18–19].

Moreover, the increased use of antifungal drugs has contributed to emerging resistance in *Candida* species which has prompted scientists worldwide to develop novel and more effective antifungal agents with a broad spectrum, better pharmacokinetic profile and low toxicity. In our previous study, we synthesized a series of novel 1,2,3-triazole derivatives from naturally bioactive alcohols and evaluated their anti-*Candida* activity as well as cytotoxicity [20]. The results showed that quinoline containing 1,2,3-triazole (**3a**) has great potential to inhibit the *Candida* spp *in vitro*. In the present work, we have extended our study further to determine the role of quinoline on anti-*Candida* activity; by replacing with its structural analogues (**3b** and **3c**). We have explored the effect of selected lead inhibitors (**3a** and **3b)** on the growth and secretion of hydrolytic enzymes in *C*. *albicans*. The plasma membrane H^+^ ATPase activity and ergosterol biosynthesis were also determined in the presence and absence of the test compounds.

## Materials and methods

### Chemistry

All the reagents and solvents purchased from Sigma-Aldrich, S.D. Fine, SRL and Hi Media, were used without further purification. Reaction was monitored by thin-layer chromatography (TLC) using pre-coated aluminum sheets (Silica gel 60 F_254_, Merck Germany) and spots were visualized under UV light. The IR spectra of the compounds were recorded on Agilent Cary 630 FT-IR spectrometer. ^1^H NMR and ^13^C NMR spectra were obtained in CDCl_3_ with reference to TMS as on internal standard using Bruker Spectrospin DPX-300 spectrometer at 300 MHz and 75 MHz respectively. Splitting patterns are designated as follows: s, singlet; d, doublet; t, triplet; m, multiplet. Chemical shift (*δ*) values are given in parts per million (ppm) and coupling constants (*J*) in Hertz (Hz). CHN analysis was recorded on Elemental Vario analyzer. Melting points were recorded on a digital Buchi melting point apparatus (M-560) and were reported uncorrected. Purification of the compounds was done by column chromatography using silica gel (230–400 mesh size) with petroleum ether/ethyl acetate (8:2) as eluent for alkynes and 10% methanol in DCM for triazole derivatives.

#### General procedure for the synthesis of alkynes (2a-c)

A round bottom flask with stir bar was charged with natural precursor (1.0 mmol) and anhydrous DMF (10 ml) and the reaction flask was cooled to 0°C. To this solution, potassium carbonate (1.0 mmol) was added, followed by slow addition of propargyl bromide (1.2 mmol). The reaction mixture was allowed to warm to room temperature and stirred overnight under argon. After completion of the reaction as confirmed by TLC, it was concentrated and water was added to the residue. The compound was extracted with ethyl acetate, dried over anhydrous sodium sulphate and concentrated under *vacuo*. The crude product was purified by column chromatography with petroleum ether: ethyl acetate (8:2) to give the desired alkynes (**2a-c**) in good to excellent yields. The synthesis of compound **2a [**8-(Prop-2-ynyloxy)quinoline] has been previously reported in our lab [20].

5-Chloro-8-(prop-2-ynyloxy)quinoline **(2b)**

Light brown crystalline solid, yield:87%, R_*f*_ (pet. ether/ethyl acetate, 8:2) = 0.48, Anal (C_12_H_8_ClNO) calc. C 66.22, H 3.70, N 6.44, found: C 66.02, H 3.47, N 6.54%. IR (neat): ν (cm^-1^) 3144, 2121, 1615, 1592, 1499, 1469, 1456, 1367, 1311, 1242, 1169, 1095, 994, 967, 931, 829, 788, 754. ^1^H NMR (300 MHz, CDCl_3_) (*δ*, ppm): 8.35 (d, 1H, *J =* 7.8 Hz, Ar-*H*), 8.21 (d, 1H, *J =* 8.1 Hz, Ar-*H*), 7.93–7.85 (m, 2H, Ar-*H*), 7.45 (d, 1H, *J =* 7.63 Hz, Ar-*H*), 5.20 (s, 2H, O*CH*_*2*_), 2.54 (s, 1H, ≡C*H*).

1-(Prop-2-ynyloxy)naphthalene **(2c)**

Brown solid, yield: 78%, R_*f*_ (pet. ether/ethyl acetate, 8:2) = 0.45, Anal (C_12_H_8_ClNO) calc. C 85.69, H 5.53, found: C 85.32, H 5.21%. IR (neat): ν (cm^-1^) 3345, 2954, 2120, 1864, 1554, 1520, 1456, 1356, 1323, 1273, 1170, 1110, 1005, 987, 845, 831, 767, 745. ^1^H NMR (300 MHz, CDCl_3_) (*δ*, ppm): 8.20 (d, 1H, *J =* 7.6 Hz, Ar-*H*), 7.97 (d, 1H, *J =* 7.5 Hz, Ar-*H*), 7.56–7.39 (m, 4H, Ar-*H*), 7.10 (d, 1H, *J =* 6.9 Hz, Ar-*H*), 5.08 (s, 2H, O*CH*_*2*_), 2.50 (s, 1H, ≡C*H*).

#### Procedure for the synthesis of benzyl azide

A mixture of benzyl bromide (1.0 mmol) and sodium azide (3.0 mmol) in anhyd. DMF (10 ml) was stirred overnight at 70°C. After completion of the reaction as monitored by TLC, the reaction was quenched with water. The crude was extracted with ethyl acetate, washed with brine, dried over anhyd. sodium sulphate and concentrated under *vacuo*. The oily residue was used as such without any further purification.

#### General procedure for the synthesis of triazole derivatives (3a-c)

Equimolar amounts of alkyne (**2a-c**) and benzyl azide were dissolved in *tert*-butanol and water (1:2) mixture. To this reaction mixture, copper sulphate (0.05 eq) and sodium ascorbate (0.01 eq) were added and stirred at room temperature till the disappearance of starting materials as indicated by TLC. The reaction mixture was quenched with saturated brine and crude was extracted with ethyl acetate, dried over anhyd. Na_2_SO_4_ and concentrated under *vacuo*. The crude was purified by column chromatography using dichloromethane and methanol (9:1) as eluent to give 1,2,3-triazole derivatives in good to excellent yields. The compound **3a** [1-Benzyl-4-(quinolin-1-yloxy)methyl)-1H-1,2,3-triazole] has been previously reported in our lab [20].

8-((1-Benzyl-1H-1,2,3-triazol-4-yl)methoxy)-5-chloroquinoline **(3b)**

Brown solid, M.pt. 148–149°C, yield: 65%, R_*f*_ (pet. ether/ethyl acetate, 5:5) = 0.15, Anal (C_19_H_15_ClN_4_O) calc. C 65.05 H 4.31 N 15.97, found: C 65.08 H 4.28 N 15.94%. IR (neat): ν (cm^-1^) 3065, 2924, 2851, 1721, 1605, 1587, 1512, 1460, 1423, 1380, 1343, 1296, 1262, 1225, 1162, 1140, 1061, 1035, 1018, 964, 855, 836, 737, 717, 678. ^1^H NMR (300 MHz, CDCl_3_) (*δ*, ppm): 8.95 (s, 1H, Ar-*H*), 8.52 (d, 1H, *J* = 8.4 Hz, Ar-*H*), 7.66 (s, 1H, triazole ring), 7.53–7.47 (m, 2H, Ar-*H*), 7.36–7.33 (m, 3H, Ar-*H)*, 7.28–7.25 (m, 3H, Ar-*H*), 5.51 (s, 2H, OC*H*_*2*_), 5.50 (s, 2H, C*H*_*2*_). ^13^C NMR (75MHz, CDCl_3_) (*δ*, ppm): 153.05, 149.76, 144.02, 140.84, 134.26, 133.05, 129.12, 128.82, 128.17, 127.10, 126.46, 123.31, 122.84, 122.33, 110.10, 63.10, 54.28.

1-Benzyl-4-((naphthalen-1-yloxy)methyl)-1H-1,2,3-triazole **(3c)**

Dark brown solid, M.pt. 128–129°C, yield: 30%, R_*f*_ (DCM/ methanol, 9:1) = 0.735, Anal (C_19_H_15_ClN_4_O) calc. C 65.05 H 4.31 N 15.97, found: C 65.08 H 4.28 N 15.94%. IR (neat): ν (cm^-1^) 3065, 2924, 2851, 1721, 1605, 1587, 1512, 1460, 1423, 1380, 1343, 1296, 1262, 1225, 1162, 1140, 1061, 1035, 1018, 964, 855, 836, 737, 717, 678. ^1^H NMR (300 MHz, CDCl_3_) (*δ*, ppm): 8.92 (s, 1H, Ar-*H*), 8.43 (d, 1H, *J* = 7.8 Hz, Ar-*H*), 7.69 (s, 1H, triazole ring), 7.41–7.32 (m, 4H, Ar-*H*), 7.27–7.24 (m, 3H, Ar-*H*), 7.05–6.98 (m, 3H, Ar-*H*), 5.56 (s, 2H, OC*H*_*2*_), 5.48 (s, 2H, C*H*_*2*_).^13^C NMR (75MHz, CDCl_3_) (*δ*, ppm): 152.27, 148.56, 143.24, 139.76, 134.35, 133.25, 129.09, 128.76, 128.17, 127.26, 126.89, 123.24, 122.72, 122.13, 110.51, 62.97, 54.24.

### *In vitro* anti-*Candida* activities

#### Determination of IC_50_ value

Broth dilution technique was employed to determine the IC_50_ values of all the synthesized 1,2,3 triazole derivatives (**3a-c**) against *C*. *albicans* ATCC 90028, C. *glabrata* ATCC 90030, *C*. *tropicalis* ATCC 750 and four clinical isolates of *C*. *albicans* namely D27, D31, D39 (FLC-sensitive) and D15.9 (FLC-resistant). The strains were maintained on yeast extract:peptone:dextrose (1:2:2:2.5) nutrient agar (2.5%) slants at 4°C. The test compounds were dissolved in DMSO with less than 4% concentration of it in the final test volume. Different concentrations (1000… 7.8 μg/mL) of test compounds in Sabouraud dextrose (SD) broth medium were dispensed into the wells, then inoculated with the test organism with approx. 2.5 × 10^3^ cells/mL and incubated at 37°C for 24 h. After incubation period, the optical density was measured at 600 nm by using Thermo Scientific Multiskan Go plate reader. The IC_50_ value was defined as the concentration of the test compound that causes 50% decrease in absorbance compared with that of the control (no test compound) [21]. All the experiments were done in triplicate at separate times.

#### Disk diffusion assay

The preliminary screening of anti-*Candida* activity showed that compound **3a** still had better inhibition in comparison to its structural analogues, **3b** and **3c**. Moreover, compound **3b** also showed significant inhibition including in FLC-resistant *C*. *albicans* strain, thus we selected compound **3a** along with **3b** as lead inhibitors. The present work is focussed on mainly *C*. *albicans* due to its pathological importance. Hence, besides one standard ATCC strain, a FLC-sensitive and FLC- resistant *C*. *albicans* clinical strains were used in the present study.

Disk diffusion assay was performed using *C*. *albicans* ATCC 90028 (standard), the two clinical isolates, *C*. *albicans* D27 (FLC-susceptible), and *C*. *albicans* D15.9 (FLC-resistant) for the lead inhibitors **3a** and **3b**. The *C*. *albicans* cells were inoculated into liquid SD medium and grown over night at 37°C. The cells were then centrifuged at 3000 rpm for 5 min, washed three times with PBS and then suspended in PBS again. Approximately, 10^5^ cells/mL were inoculated into molten SD agar medium at 42°C and poured into 100-mm-diameter petriplates. Sterilized 4 mm disks of Whatman paper were placed on solid agar and 1, 2, and 4 μL of **3a** and 2, 4, and 8 μL of **3b** from the stock solution of 10 mg/mL were spotted on the disk. 2 μL of FLC from the stock of 10 mg/mL was also applied on the disks to serve as positive control. The diameter of zone of inhibition (ZOI) was recorded in millimetres after 48 h and was compared with that of control. This experiment was repeated thrice and values were shown in terms of mean ±standard error of mean.

#### Growth assay

The *C*. *albicans* cells were freshly revived by subculture on the SD agar plate. A loopful of inoculum was introduced into the SD broth and cells were grown for 16 h at 37°C before use. Approximately 2×10^3^ cells/mL were then inoculated into the freshly prepared 50 mL sterile SD medium. Different concentrations, equivalent to 2MIC, MIC, MIC/2, of test compounds were added separately into the conical flasks containing inoculated medium and incubated at 37°C and 160 rpm. Strain specific concentration of FLC was used as positive control *viz* 40 μg/mL for *C*. *albicans* ATCC 90028, 20 μg/mL for *C*. *albicans* D27 (FLC-susceptible) and 40 μg/mL for *C*. *albicans* D15.9 (FLC-resistant). At predetermined time periods (0, 2, 4, 6, 8, 10, 12, 14, 16, 18, 20 22, and 24 after incubation with agitation at 37°C), 1 mL aliquot of each sample was removed from the conical flask and growth was measured tubidometrically at 600 nm using Thermo Multiskan spectrophotometer. Optical density was recorded for each concentration against time (h).

#### Time kill curve study

Time-kill curves were used for testing the fungicidal or fungistatic nature of the lead inhibitors **3a** and **3b**. The experimental procedure was performed according to the method reported by Wang et al. [22]. *C*. *albicans* ATCC 90028, *C*. *albicans* D27 (FLC-susceptible) and *C*. *albicans* D15.9 (FLC-resistant) strains were used as test organisms. Fluconazole was used as a positive control. Freshly prepared sterile YEPD medium was inoculated by fresh culture (approx. 2×10^3^ cells) of test organisms in different conical flasks. The cells were exposed to the test compounds at 2MIC, MIC and MIC/2 values, separately. All the conical flasks having different sets of test compounds and concentrations were incubated at 37°C and 160 rpm. At predetermined time points (0, 4, 12, 24, and 48 h after incubation with agitation at 37°C), a 100-μL aliquot was removed from every solution and appropriately diluted in sterile water. A 100-μl aliquot from each dilution was spread over the YEPD agar plate. Colony counts were determined after incubation at 37°C for 24 h. All the time-kill curve experiments were conducted in duplicate and mean colony count data (log_10_ CFU/mL) were plotted as a function of time for each strain.

### Effect on hydrolytic enzyme secretion in *C*. *albicans*

As described previously, secretion of hydrolytic enzymes (proteinases and phopholipases) play an important role in the pathogenicity of *Candida* spp. In the present study we have determined the effect of lead compounds **3a** and **3b** on the secretion of proteinases and phopholipases.

#### Proteinase assay

*C*. *albicans* ATCC 90028, *C*. *albicans* D27 (FLC-susceptible) and *C*. *albicans* D15.9 (FLC-resistant), previously identified as proteinase positive *C*. *albicans* [23] were transferred to flasks containing 5mL YEPD media and incubated at 37°C for 18 h. Following incubation, 1.5 mL of the yeast culture was taken into micro centrifuge tubes and centrifuged at 3000 rpm, for 5 min. The pellet obtained was washed by phosphate buffer saline (PBS) twice to remove the residual medium. After standardizing the suspensions (MacFarland standard), cells were exposed for 1 h to MIC and MIC/4 concentration of lead inhibitors **3a** and **3b**. Volumes of 1μL were placed at equidistant points on proteinase agar medium (0.1% yeast nitrogen base w/o amino acids; 0.145% ammonium sulfate; 2% glucose with 0.2% (w/v) BSA fraction V mixed into agar at ∼40°C) and incubated at 37°C for 3 days [24]. The experiment was performed in duplicate. Secreted proteinase enzyme will degrade BSA and form zone of clearance around the yeast colonies. An index (Pz value) was used to represent the extent of proteinase activity by different strains of *C*. *albicans*. A Pz value of 1.0 depicts no enzyme activity. It was calculated as follows:

$$Pz\;value=\frac{diameter\;of\;colony}{diameter\;of\;colony+diameter\;of\;zone\;of\;degradation}$$

#### Phospholipase assay

Phospholipase activity was performed using the method of Price et al. (1982) with minor modifications [25]. The cells were incubated at 37°C for 18 h and then centrifuged at 3000 rpm for 5 min. The pellet obtained was washed with PBS twice to remove the residual culture medium. After standardizing the suspension, cells were exposed for 1 h to the test compounds at MIC and MIC/4. Volumes of 1 μL were placed at equidistant points on phospholipase agar media plates (1% peptone; 3% glucose; 5.73% NaCl; 0.055% CaCl_2_ with 10% (v/v) egg yolk emulsion) and incubated at 37°C for 4 days. The secretion of phospholipases was determined by the formation of opaque zones due to precipitation around the yeast colonies. The assay was conducted in duplicate. Pz values were calculated as follows:

$$Pz\;value=\frac{diameter\;of\;colony}{diameter\;of\;colony+diameter\;of\;zone\;of\;precipitaion}$$

### Proton extrusion measurement

The proton pumping activity of *C*. *albicans* was determined by monitoring acidification of external medium by measuring pH as describe earlier [17]. In brief, the mid-log phase cells were harvested from SD medium by centrifugation at 3000 rpm for 15 min which is followed by three washings with phosphate buffer saline (PBS). A solution containing 0.1 M KCl and 0.1 mM CaCl_2_ in distilled water was prepared. Compounds **3a** and **3b** at the concentrations of 50 and 100 μg/mL were separately added in this solution. Six sets of solution (10 mL) were prepared *viz* two having 50 and 100 μg/mL of **3a**, two having 50 and 100 μg/mL of **3b**, one having 10 μg/mL of FLC (as standard drug) and one is without test compound (control). Initial pH was adjusted to 7.0 using 0.01 M HCl/NaOH. Approximately 0.2 g of cells were suspended in each set of solution. Suspension was kept in a double-jacketed glass container with constant stirring. Again, the pH of suspension was maintained as 7.0 for 1 min by using 0.01 N NaOH. The rate of H^+^ extrusion was then monitored and calculated from the volume of 0.01 N NaOH consumed per min per mg weight of cells. Glucose has been reported as the stimulator of proton extrusion pump [17, 26]. Therefore above experiment was also repeated in the presence of 5 mM of glucose.

### Measurement of intracellular pH (pHi)

In yeast cells, plasma membrane H^+^-ATPase helps to maintain internal pH between 6.0 and 7.5. Intracellular pH (pHi) was measured as reported earlier by Manzoor et al. [17]. Mid-log phase cells were harvested from SD medium and washed thrice with PBS. Subsequently, cells (0.1 g) were suspended in 5 mL solution of 0.1 M KCl, and 0.1 mM CaCl_2_ in distilled water. Compounds **3a** and **3b** were added to the suspension at a desired concentration of 100 μg/mL. 10 μg/mL of standard drug FLC was added to a separate suspension for comparison. Initial pH of the suspension was adjusted to 7.0 in each case and incubated for 30 min at 37°C with constant stirring. After incubation, the pH of suspension was again adjusted to 7.0 followed by the addition of nystatin (20 μM) and incubation at 37°C for 1 h. The change in pH of the suspension was monitored on a pH meter with constant stirring. The value of external pH at which nystatin permeabilization induced no further shift was taken as an estimate of pHi. Nystatin gives pHi values close to the cytoplasmic pH values, as it does not affect the mitochondria [27].

### Transmission electron microscopic analysis

The morphology of *C*. *albicans* ATCC 90028 was analyzed using TEM following the standard protocol [28]. Mid-log phase cells were harvested, standardized (A600 ≈ 0.1) and exposed to MIC of lead inhibitors **3a** and **3b** for 1 h. Cells were then washed thrice with phosphate buffer solution to remove the residual medium and fixed overnight in 2.5% glutaraldehyde in phosphate/magnesium buffer (40 mM K_2_HPO_4_/KH_2_PO_4_, pH 6.5–0.5 mM MgCl_2_). Cells were washed twice for 15 min in 0.1 M sodium phosphate buffer (pH 6.0) and post-fixed for 2 h in 2% osmium tetroxide. Cells were again washed twice for 15 min in distilled water and then en bloc stained with 1% uranyl acetate (aqueous) for 30 min. After two further washes, cells were dehydrated in 95% and 100% ethanol. Cells were then exposed to propylene oxide for 2×10 min and infiltrated for 1 h in 1:1 propylene/epoxy embedding material (Epon) mixture and then overnight in fresh Epon. After polymerization for 48 h at 60°C, ultrathin sections were cut using a microtome (LeicaEM UC6) and transferred to a copper grid. Samples were stained with uranyl acetate (saturated solution of uranyl acetate in 50% alcohol) followed by lead citrate. Samples were washed three times in Milli-Q (MQ) water and dried by touching Whatman filter paper. Sections were examined with a FEI Tecnai G^2^ TF20 transmission electron microscope at 200 KV.

### Ergosterol estimation assay

The total intracellular sterols were extracted as reported earlier with slight modifications [29]. As previous studies showed almost similar behavior of standard and FLC-sensitive strains of *C*. *albicans*, in the present study we used only standard *C*. *albicans* ATCC 90028 and compared with a FLC-resistant strain. Three separate conical flasks were used containing compounds **3a** and **3b** at MIC, MIC/2 and MIC/4 in SD broth inoculated with freshly cultured cells of *C*. *albicans* ATCC 90028 (standard) and FLC-resistant *C*. *albicans*. Fluconazole (40 μg/mL) was used as a positive control while untreated cells as negative control for comparison. All the conical flasks were incubated at 35°C for 16 h. After incubation, the cells were harvested at their stationary phase and the weight of pellet was determined. The pellet was treated with 25% alcoholic potassium hydroxide (KOH) solution followed by incubation at 85°C for 1 h. After incubation, sterol were extracted by the addition of *n*-heptane:distilled water (1:3). The heptane layers were transferred in fresh test tube, diluted five-fold in 100% ethanol and scanned spectrophotometrically between 230 and 300 nm. The presence of ergosterol and the late sterol intermediate 24(28) DHE in the extracted samples resulted in a characteristic four peak curve. The absence of detectable ergosterol in the extract was indicated as a flat line. Ergosterol content was calculated as percentage wet weight of the cells by the following equation:

$$\%\;Ergosterol+\%\;24(28)DHE=[(A_{281.5}÷290)\times F]÷pellet\;weight$$

here, % 24(28)DHE = [(A_230_/518)×*F*]/pellet weight, A_281.5_ and A_230_ absorbance at 281.5 nm and 230 nm, respectively and *F* is the dilution factor in alcohol.

### Cytotoxicity

Cytotoxicity of compounds **3a** and **3b** was performed by hemolytic assay and cell proliferation assay.

#### Hemolytic assay

The hemolytic activities of the test compounds **3a** and **3b** along with standard drug FLC were determined on human red blood cells (hRBCs) [30]. Human blood from a healthy individual was collected in tubes containing EDTA as anti-coagulant. The erythrocytes were harvested by centrifugation for 10 min at 2000 rpm and 20°C, washed three times in PBS. To the pellet, PBS was added to yield a 10% (v/v) erythrocytes/PBS suspension. The 10% suspension was then diluted 1:10 in PBS. From each suspension, 100 μL was added in triplicate to 100 μL of a different dilution series of test compounds in the same buffer in micro centrifuge tubes. Total hemolysis was achieved with 1% Triton X-100. The tubes were incubated for 1 h at 37°C and then centrifuged for 10 min at 2000 rpm and 20°C. From the supernatant, 150 μL was transferred to a flat-bottomed microtiter plate (Tarson), and the absorbance was measured spectrophotometrically at 450 nm. The hemolysis percentage was calculated by following equation:

$$\%\;Hemolysis=[(A_{450}\;of\;test\;compound\;treated\;sample-A_{450}\;of\;buffer\;treated\;sample)÷(A_{450}\;of\;1\%\;Triton\;X-100\;treated\;sample-A_{450}\;of\;buffer\;treated\;sample)]\times 100$$

#### Cell proliferation assay

The *in vitro* cytotoxicity of compounds **3a** and **3b** to mammalian cells was also determined. The assay was performed in 96-well tissue culture-treated plates as described earlier [31]. Vero cells (monkey kidney fibroblasts) were seeded to the wells of 96-well plate at a density of 25,000 cells/well and incubated for 24 h. Samples at different concentrations were added and plates were again incubated for 48 h. The number of viable cells was determined by Neutral Red assay. IC_50_ values were obtained from dose response curves. Doxorubicin was used as a positive control for cytotoxicity.

## Results

### Chemistry

**Fig 1:**
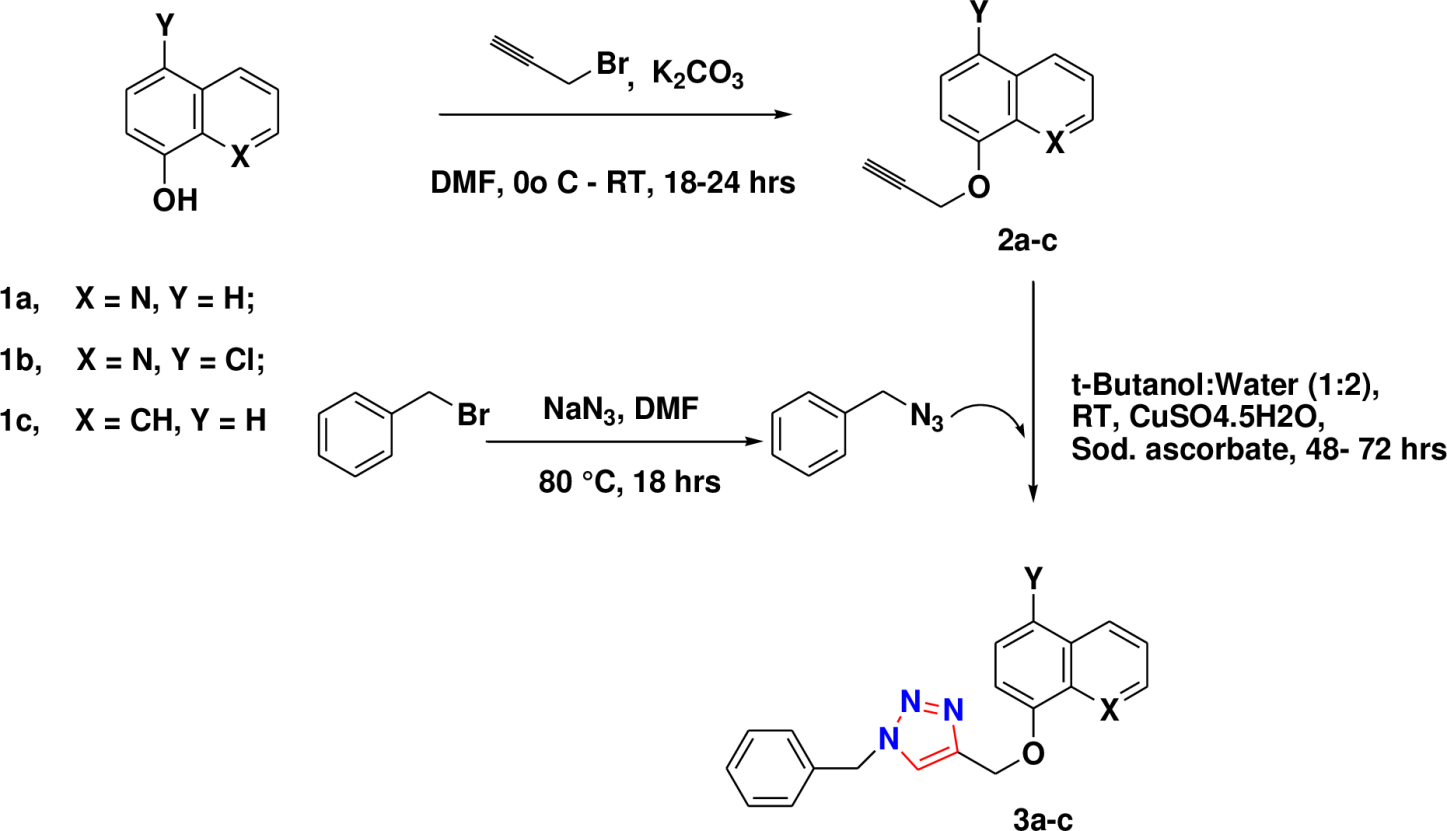
Synthetic route of 1,2,3-triazole compounds (3a-c). 8-hydroxy quinoline (1a), 5-Cl- 8-hydroxy quinoline (1b) and 1-naphthol (1c) were used as precursor compounds which converted into 1,2,3 tiazoles.

The two structural analogues (**3b** and **3c**) of quinoline containing 1,2,3-triazole (**3a**) were synthesized as shown in Fig 1. The precursor alcohols were converted into 1,2,3-triazoles via copper catalyzed [3+2] azide-alkyne cycloaddition reaction (S1 and S2 Figs).

### Biological studies

Previously, we reported the synthesis and anti-*Candida* activities of eight novel 1,2,3 triazole derivatives (**3a-h**) and found that compound **3a** displayed better activity than its natural bioactive compound 8-hydroxy quinoline. This compound showed an IC_50_ of 0.044, 12.02 and 3.60 μg/mL against *C*. *albicans*, *C*. *glabrata* and *C*. *tropicalis*, respectively. This enhanced anticandidal activity of compound **3a** may be due to the presence of quinoline ring along with 1,2,3 triazole ring in its structure [20]. To determine the role of *N*-atom present in the quinoline ring and the effect of substitution on quinoline ring, we synthesized two new structural analogues (**3b** and **3c**). Compound **3b** with chlorine substitution on quinoline ring showed good anticandial activity with IC_50_ value of 25.4, 73.3 and 23.6 μg/mL against *C*. *albicans*, *C*. *glabrata* and *C*. *tropicalis*, respectively whereas compound **3c** having naphthalene instead of quinoline ring showed slightly less activity with IC_50_ values of 91.2, 106.9 and 38.2 μg/mL against *C*. *albicans*, *C*. *glabrata* and *C*. *tropicalis*, respectively. The results hence indicate that *N*-atom plays an important role in the anticandidal activity and quinoline is a key moiety along with 1,2,3 triazole ring in the structure of these synthesized compounds. Hence compound **3a** and **3b** were selected for further evaluation of the biological activity of these compounds. Moreover, chlorine substitution in compound **3b** might be the reason for its increased activity but compound **3a** was still found to be the most potent anticandidal agent with lower IC_50_ values (Table 1).

**Table 1:** Anti-*Candida* activity of newly synthesized 1, 2, 3-triazoles.

Compound	Anti-*Candida* Activity (IC_50_± S.D. in μg/mL)						
	*C*. *albicans ATCC90028*	*C*. *glabrata ATCC90030*	*C*. *tropicalis ATCC750*	*C*. *albicans* D27	*C*. *albicans* D31	*C*. *albicans* D39	*C*. *albicans* D15.9
**3a**	0.044 ± 0.11	12.02 ± 0.02	3.60 ± 0.07	1.9± 0.09	3.3±0.065	4.1±0.02	2.3±0.05
**3b**	25.4± 0.76	73.3± 1.41	23.6± 0.83	33.7±1.51	41.5±1.24	47.1±0.39	32.8±1.25
**3c**	91.2± 0.76	106.9 ± 1.41	38.2 ± 0.83	43.7±1.18	118.9±2.94	62.2±1.07	66.9±1.84
**FLC**	15.62 ± 0.65	7.81 ± 0.88	8.55 ± 1.59	<7.5±0.00	<7.5±0.00	<7.5±0.00	>1000±0.0

*C*. *albicans* D27, *C*. *albicans* D31, *C*. *albicans* D39 = clinical isolates of *C*. *albicans* (FLC sensitive); *C*. *albicans* D15.9 = clinical isolates of *C*. *albicans* (FLC resistant); FLC = Fluconazole.

The anticandial activity of compounds **3(a-c)** was determined for clinical *Candida* isolates also besides the standard strains. Three FLC susceptible strains (*C*. *albicans* D27, *C*. *albicans* D31 and *C*. *albicans* D39) and one FLC resistant strain (*C*. *albicans* D15.9) were used in this study. Again, compound **3a** showed best inhibition with IC_50_ values of 1.9, 3.3, 4.1 and 2.3 μg/mL against *C*. *albicans* D27, *C*. *albicans* D31, *C*. *albicans* D39 and *C*. *albicans* D15.9 strains, respectively. Among the structural analogues of **3a**, **3b** showed better anticandidal results than **3c** with IC_50_ values of 33.7, 41.5, 47.1 and 32.8 μg/mL against *C*. *albicans* D27, *C*. *albicans* D31,*C*. *albicans* D39 and *C*. *albicans* D15.9, respectively as compared to 43.7, 118.9, 62.2 and 66.9 μg/mL in case of compound **3c**, respectively (Table 1). Moreover, both the test compounds **3a** (MIC 25 μg/mL) and **3b** (MIC 250 μg/mL) showed many fold increased activity against FLC resistant strain in comparison to FLC (MIC >1 μg/mL). On the basis of these preliminary results, compounds **3a** and **3b** were selected for further biological evaluation. It is well known that 50–60% cases of candidiasis occur due infection with *C*. *albicans* [32]. Although, non-*albicans* species are also important, our study here is focused mainly on the *C*. *albicans* strains.

*C*. *albicans* ATCC 90028 (standard), clinical isolates *C*. *albicans* D27 (FLC-susceptible) and *C*. *albicans* D15.9 (FLC-resistant) were grown on solid agar plates and exposed to the various concentrations of lead inhibitory compounds (**3a** and **3b**) as well as FLC. All the strains, including the FLC-resistant, showed higher susceptibility to both the test compounds, but **3a** showed better results. Whatman filter disks, impregnated with 40 μg of **3a** showed 39, 26 and 32 mm ZOI for *C*. *albicans* ATCC 90028 (standard), *C*. *albicans* D27 (FLC-susceptible) and *C*. *albicans* D15.9 (FLC-resistant), respectively. Similarly, the diameters of the ZOI around the disks impregnated with 80 μg of compound **3b** were measured to be 29, 26, and 22 mm for *C*. *albicans* ATCC 90028 (standard), *C*. *albicans* D27 (FLC-susceptible) and *C*. *albicans* D15.9 (FLC-resistant), respectively. Compound **3a** displayed much better inhibition in comparison to FLC against the standard and FLC-resistant strains. At the same concentration, compound **3a** showed ZOI of 35 and 26 mm for *C*. *albicans* ATCC 90028 (standard) and *C*. *albicans* D15.9 (FLC-resistant) respectively, while FLC showed ZOI of 26 and 24 mm for the same strains (S3 Fig). The zone formed around the disks impregnated with compound **3a** were clear which indicating towards its potential fungicidal nature while compound **3b** and FLC showed turbid zones which indicated towards their fungistatic nature. The results are summarised in Table 2.

**Table 2:** Antifungal susceptibility using disk diffusion assay for the lead compounds 3a and 3b against *C*. *albicans* ATCC 90028, FLC-susceptible *C*. *albicans* D27 and FLC-resistant *C*. *albicans* D15.9.

Compound (μg)	Zone of Inhibition (mm)		
	*C*. *albicans ATCC 90028* (Standard)	*C*. *albicans* D27(FLC-susceptible)	*C*. *albicans* D15.9(FLC-resistant)
**3a** (40 μg)	39± 1.12	26± 0.92	32± 1.28
**3a** (20 μg)	35± 0.97	19± 0.95	26± 073
**3a** (10 μg)	28± 0.72	14± 0.70	20± 0.21
**3b** (80 μg)	29± 0.93	26± 1.17	22± 0.79
**3b** (40 μg)	25± 0.90	18± 0.87	18± 0.09
**3b** (20 μg)	18± 0.72	16± 0.54	15± 0.21
**FLC** (20 μg)	26± 1.02	27± 1.35	24± 0.74

**Fig 2:**
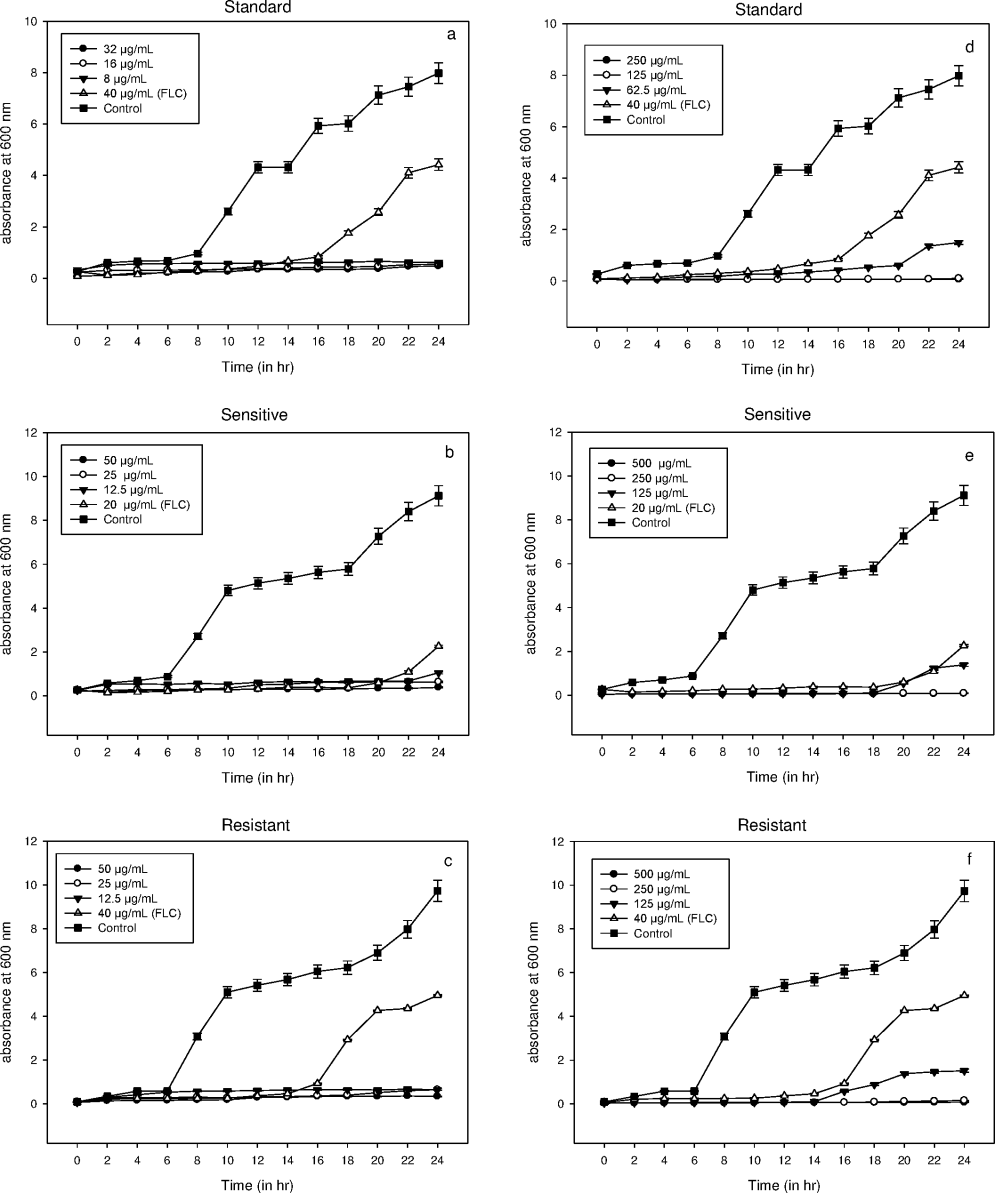
(a-f). Dose dependent growth curve of a) *C*. *albicans* ATCC 90028 b) FLC-susceptible *C*. *albicans* D27 and c) FLC-resistant *C*. *albicans* D15.9 in the presence compound 3a and d) *C*. *albicans* ATCC 90028 e) FLC-susceptible and f) FLC-resistant *C*. *albicans* in the presence of compound 3b. Growth was observed in the presence of test compounds at 2MIC, MIC and MIC/2 and was significantly inhibited at all concentrations. No growth was observed when cells were exposed to compound 3a with the lag phase extending beyond 24h. Error bars represent Mean±S.D. from three independent recordings.

The life cycle of microbial cells is completed in four phases *i*.*e*. lag, log or exponential, stationary and decline phase. The growth of *C*. *albicans* ATCC 90028 (standard), clinical isolates *C*. *albicans* D27 (FLC-susceptible) and *C*. *albicans* D15.9 (FLC-resistant) was observed in the presence of compounds **3a** and **3b** at 2MIC, MIC and MIC/2. The untreated cells showed a lag phase of 6–8 h in all the three strains. The cells did not show any growth when exposed to compound **3a** with a continuous lag phase of more than 24 h. However, at 40 μg/mL of FLC, lag phase was over by 16, 20 and 16 h in case of *C*. *albicans* standard, FLC-susceptible and FLC-resistant, respectively (Fig 2). Compound **3b** also showed significant growth inhibition at 2MIC and MIC. At the sub-MIC values of compound **3b,** growth was observed after 20, 18 and 14 h for *C*. *albicans* standard, FLC-susceptible and FLC-resistant, respectively. Thus, **3a** showed high potential as an inhibitor of *Candida* cell growth even at sub-MIC concentrations. Moreover, compound **3b** proved to be a good antifungal when compared to the standard drug FLC as an inhibitor of *Candida* cell growth.

**Fig 3:**
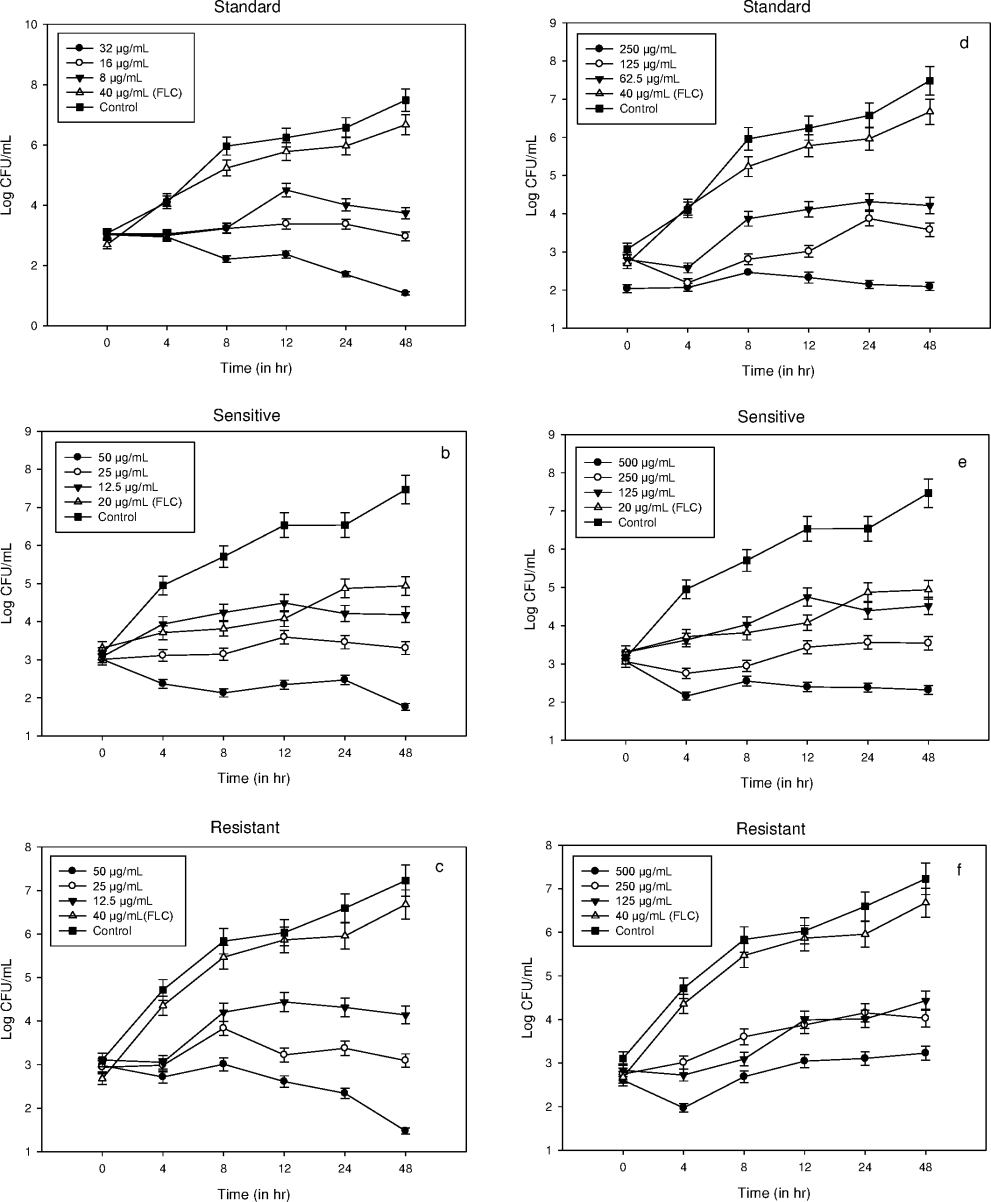
(a-f). Representative time kill curves of (a) *C*. *albicans* ATCC 90028 (b) FLC-susceptible *C*. *albicans* D27 and (c) FLC-resistant *C*. *albicans* D15,9 in the presence compound 3a and (d) *C*. *albicans* ATCC 90028 (e) FLC-susceptible and (f) FLC-resistant *C*. *albicans* in the presence of compound 3b. The results suggest that compound 3a has fungicidal nature while compound 3b is fungistatic. Error bars represent Mean±SD from three independent recordings.

Fungistatic or fungicidal nature of lead inhibitory compounds (**3a** and **3b)** was determined by time kill curve studies on *C*. *albicans* ATCC 90028 (standard) and the two clinical isolates of *C*. *albicans* (FLC-susceptible and resistant). At 2MIC, compound **3a** was fungicidal whereas compound **3b** was fungistatic as also observed in the growth curves. However, no complete fungicidal point was obtained for the compound **3a** at 2MIC, but significant decrease in CFU/mL was observed after 48 h in all the three test strains. There was no significant difference in CFU/mL observed for the untreated and FLC (40 μg/mL) treated cells of *C*. *albicans*, both standard and FLC-resistant. As discussed earlier, compound **3b** showed fungistatic behaviour as only slight increase in CFU/mL was recorded with time interval. However, this increase was dose dependent and significant inhibition was observed at higher concentrations. Moreover, both the test compounds showed better inhibition in comparison to the standard drug FLC (Fig 3).

**Fig 4:**
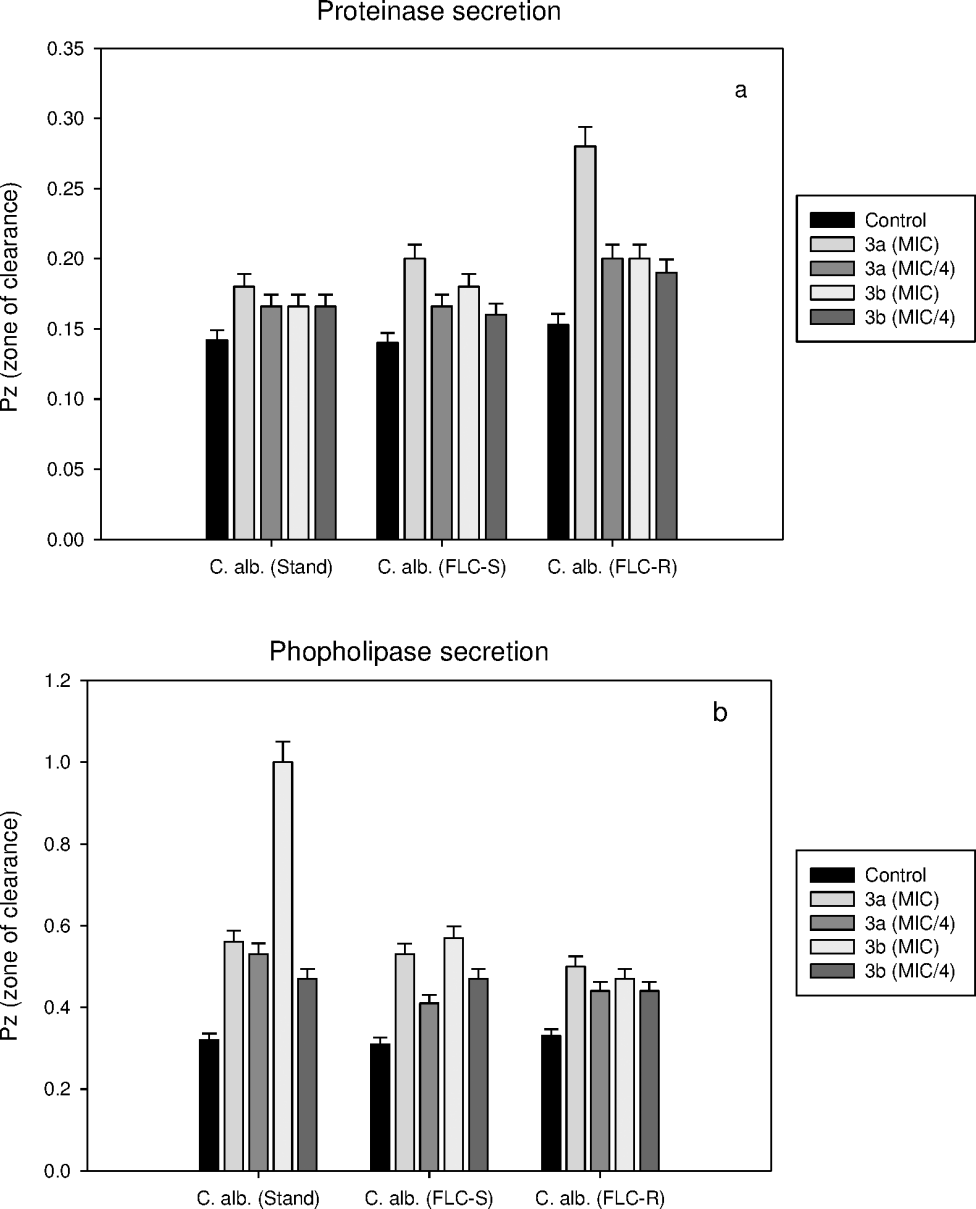
(a-b). Effect of compounds 3a and 3b on a) proteinase and b) phospholipase secretion by three strains of C. *albicans*. Pz value is the ratio of the diameter of colony to the diameter of colony plus zone of degradation/precipitation. High Pz value represents low enzyme activity. Compound 3a decreased proteinase secretion by two fold in FLC-resistant *C*. *albicans* D15.9. No phospholipase activity was observed in *C*. *albicans* ATCC 90028 after treatment with compound 3b. Error bars represents Mean±S.D. from three independent recordings.

The test compounds (**3a** and **3b)** were further explored to see their effects on the secretion of hydrolytic enzymes, proteinases and phospholipases. Although no significant inhibition of proteinase secretion was observed in the presence of either compound, yet compound **3a** showed 45% inhibition of proteinase secretion in FLC-resistant strain of *C*. *albicans* at MIC. Results showed that proteinase secretion decreased by 21, 30 and 45% when treated with MIC of compound **3a** and 14, 22 and 23.5% when treated with the MIC of compound **3b** in *C*. *albicans* ATCC 90028 (standard), *C*. *albicans* D27 (FLC-susceptible) and *C*. *albicans* D15.9 (FLC-resistant), respectively. A slight decrease in % inhibition was observed when treated with lead compounds at MIC/4. Similarly, phospholipase secretion decreased by 43, 42 and 34% when treated with MIC of compound **3a** and 68, 46 and 30% when treated with the MIC of compound **3b** in *C*. *albicans* ATCC 90028 (standard), *C*. *albicans* D27 (FLC-susceptible) and *C*. *albicans* D15.9 (FLC-resistant), respectively. At lower concentrations (MIC/4), the decrease in phospholipase secretion was 40, 25 and 25% when treated with compound **3a** and 32, 34 and 25% when treated with the compound **3b** in standard, FLC-susceptible and FLC-resistant *C*. *albicans*, respectively. The results indicated that *C*. *albicans* D15.9 (FLC-resistant) is more sensitive to proteinase secretory activity of the fungus in comparison to phospholipase secretion when treated with compound **3a**. The results are shown in (Fig 4).

The fungal plasma membrane H^+^ ATPase is a crucial enzyme that regulates intracellular pH and nutrient uptake into the cell. The process of proton extrusion is a phenomenon associated with enzyme activity which is stimulated in presence of glucose [33]. The inhibition of this protein causes the intracellular acidification which may result in cell death. The effect of the lead inhibitory compounds (**3a** and **3b)** was investigated on the activity of H^+^ ATPase enzyme by determining the rate of H^+^ extrusion in the extracellular medium. The rate of H^+^ extrusion was calculated in μmoles min^−1^mg^−1^ *Candida* cells by titrating the cell suspension with 0.01N NaOH. H^+^ extrusion rate was also determined in presence of glucose which acts as a stimulator of H^+^ efflux pump. The results are summarized in Table 3. At 100 μg/mL, compound **3a** showed 70, 45, and 40% inhibition of H^+^ extrusion in *C*. *albicans* ATCC 90028 (standard), clinical isolates *C*. *albicans* D27 (FLC-susceptible) and *C*. *albicans* D15.9 (FLC-resistant), respectively. Similarly, at the same concentration, **3b** showed 47, 65, and 42% inhibition of H^+^ extrusion in *C*. *albicans* ATCC 90028 (standard), *C*. *albicans* D27 (FLC-susceptible) and *C*. *albicans* D15.9 (FLC-resistant), respectively. Both the compounds showed greater inhibition than standard drug FLC with 38, 49 and 25% inhibition of H^+^ extrusion in *C*. *albicans* standard, FLC-susceptible and FLC-resistant, respectively. In the presence of 5 mM of glucose, the rate of H^+^ extrusion increased by 33, 24 and 36% in the standard), FLC-susceptible and FLC-resistant *C*. *albicans* strains, respectively. Again, in the presence of glucose, 52, 52 and 54% inhibition by compound **3a** and 64, 52 and 52% inhibition by compound **3b** was calculated as H^+^ extrusion rate in the standard, FLC-susceptible and FLC-resistant strains, respectively. At low concentrations of the compounds, the inhibition of H^+^ extrusion rate decreased (S4 Fig).

**Table 3:** Effect of 3a and 3b on the rate of H^+^ efflux by *C*. *albicans* ATCC 90028, FLC-susceptible *C*. *albicans* D27 and FLC-resistant *C*. *albicans* D15.9 in the presence and absence of glucose (G). Both the test compounds had a greater inhibitory effect on H^+^ efflux in comparison to FLC.

Condition	Range of relative H^+^efflux rate (×10^−6^ mol min^−1^ mg cells^−1^)		
	*C*. *albicans* ATCC 90028	*C*. *albicans* D27(FLC-susceptible)	*C*. *albicans* D15.9(FLC-resistant)
Control	36.65 ± 1.8	30.30 ± 1.5	27.80 ±1.3
**3a** (100 μg/mL)	11.00 ± 0.5	16.60 ± 0.8	16.70 ± 0.8
**3a** (50 μg/mL)	19.60 ± 0.9	20.00 ± 1.0	21.60 ± 1.0
**3b** (100 μg/mL)	19.30 ± 0.9	16.00 ± 0.8	16.00 ± 0.8
**3b** (50 μg/mL)	23.40 ± 1.1	17.60 ± 0.8	18.30 ± 0.9
Glucose (G) (5mM)	54.95 ± 2.7	40.00 ± 2.0	43.33 ± 2.1
G + **3a** (100 μg/mL)	26.60 ± 1.3	19.30 ± 0.9	20.00 ± 1.0
G + **3a** (50 μg/mL)	33.40 ± 1.6	21.60 ± 1.0	28.30 ± 1.4
G + **3b** (100 μg/mL)	20.00 ± 1.0	19.30 ± 0.9	21.00 ± 1.0
G + **3b** (50 μg/mL)	23.30 ± 1.1	22.30 ± 1.1	25.00 ± 1.2
FLC(10 μg/mL)	22.85 ± 1.1	15.35 ± 0.7	20.95 ± 1.0

**Fig 5:**
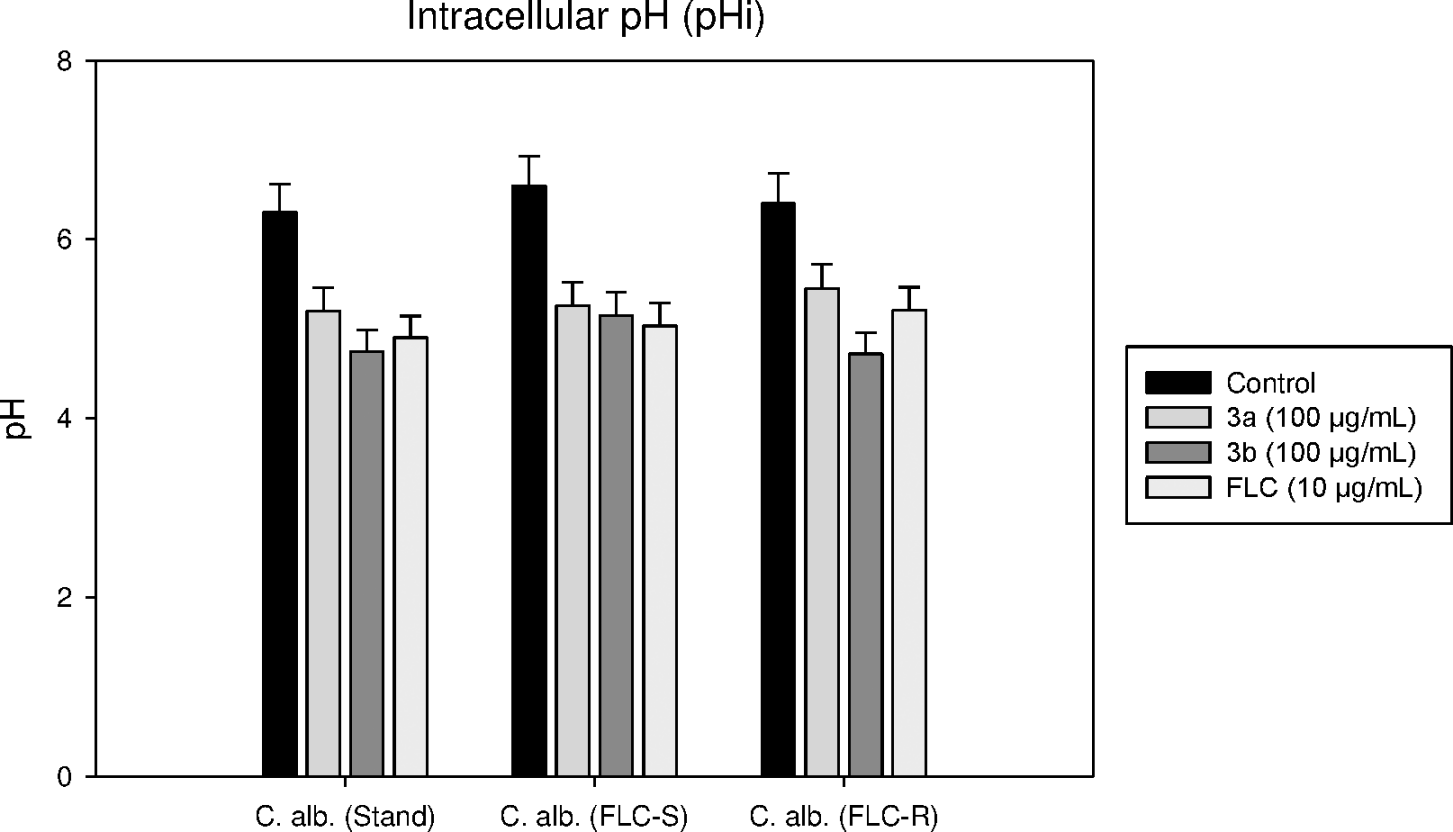
Intracellular pH of *C*. *albicans* ATCC 90028; FLC-susceptible *C*. *albicans* D27and FLC-resistant *C*. *albicans* D15.9 with and without the treatment of 100 μg/mL of test compounds 3a and 3b. Fluconazole (10 μg/mL) was used as standard drug. The decrease in pH showed the accumulation of H^+^ ions due to reduced activity of the proton pump. The acidity error bars represent Mean±S.D. from three independent recordings.

The enzyme activity of H^+^ ATPase can also be determined by evaluating intracellular pH of *Candida* cells. The intracellular pH of these cells is maintained within the range of 6.0–7.5 due to the activity of H^+^ ATPase. The inhibition of this protein blocks the permeability of H^+^ ions across plasma membrane which results in the alteration of pH inside the cell [17]. Here we studied the change in pHi in the presence of **3a** and **3b** which is, as mentioned earlier, directly related to H^+^ ATPase enzyme activity. In case of *C*. *albicans* ATCC 90028, the intracellular pH reduced from 6.3 to 5.2 and 4.7 in presence of compound **3a** and **3b**, respectively, while in *C*. *albicans* D27 (FLC-susceptible) it reduced from 6.6 to 5.2 and 5.1, respectively. Similarly, in *C*. *albicans* D15.9 (FLC-resistant), pH reduced from 6.4 to 5.4 and 4.7 in presence of compound **3a** and **3b**, respectively. The results are shown in Fig 5.

**Fig 6:**
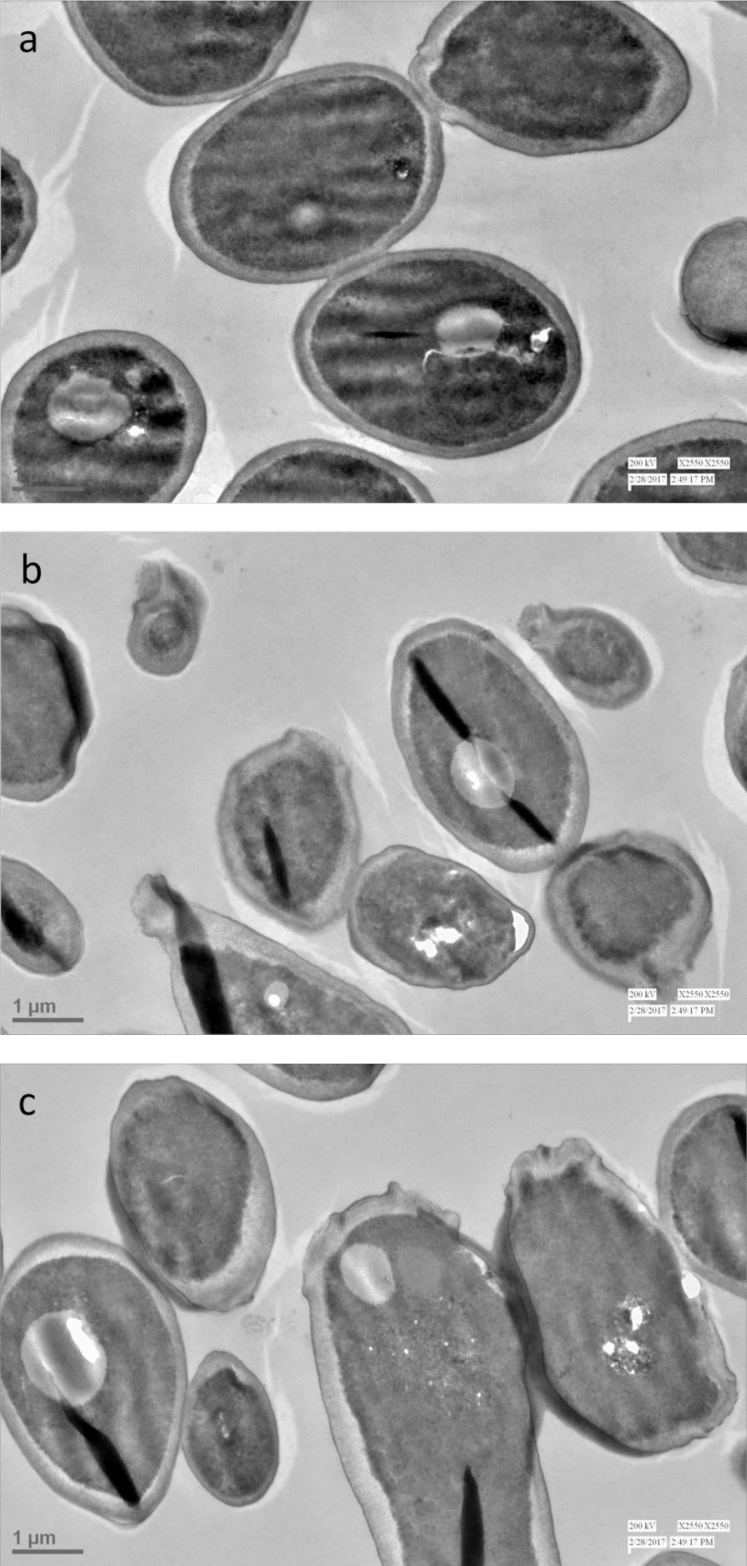
(a-c). TEM images of *C*. *albicans* a) untreated cells; b) treated with compound 3a; and c) treated with compound 3b. The morphological characteristics of untreated cells are clearly distinct from cells treated with compound 3a and 3b. (scale bar = 1μm)

The effect of lead compounds (**3a** and **3b**) on the morphology of *C*. *albicans* was monitored by transmission electron microscopy (TEM) to explore their possible mechanism of action. *Candida* cells treated with test compounds at their respective MICs and untreated control cells were used for TEM analysis. It was observed, as shown in Fig 6, that untreated cells are normal in shape with uniform cell density and intact cell membrane while treated cells showed several deformations. In case of exposed cells significant damage could be seen wherein the cell shape became abnormal and the cell membrane lost its integrity becoming irregular. It is well known that azole drugs like FLC inhibit ergosterol biosynthesis destroying both membrane integrity and function of some membrane-bound proteins and therefore affecting fungal cell growth and proliferation. Thus, we can conclude from the results of TEM analysis that the lead compounds (**3a** and **3b)** may be interfering with biosynthesis of cell membrane. The result of phase contrast microscopy (phase plate 3, Nikon Eclipse 80i) also showed the clear morphological differences between treated and untreated cells (S5 Fig).

**Fig 7:**
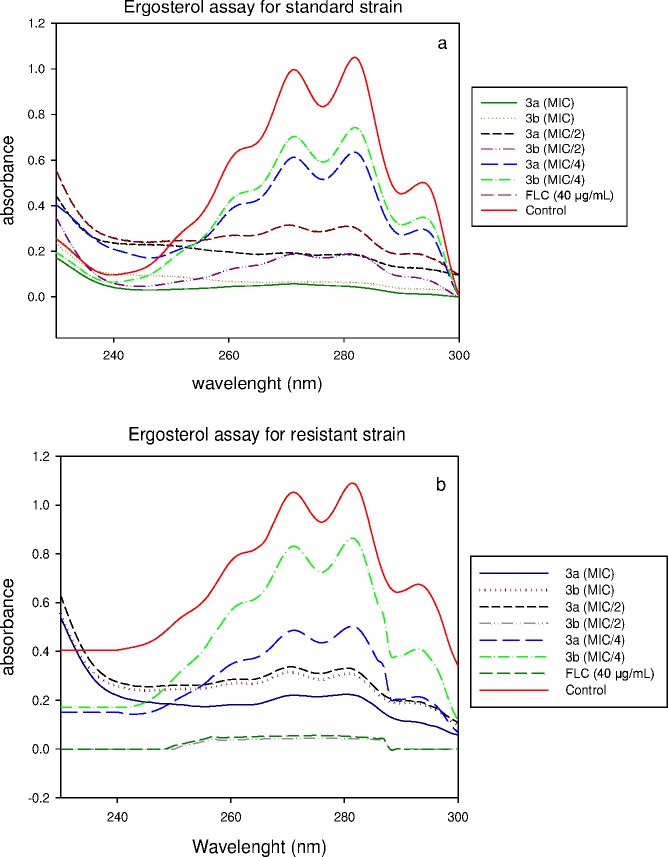
UV spectrophotometric sterol profiles of (a) *C*. *albicans* ATCC 90028and (b) FLC-resistant *C*. *albicans* D15.9 after treatment with the test compounds 3a and 3b. Strains were grown for 16 h in SD broth containing various concentrations of test compounds (MIC/4, MIC/2, MIC). Sterols were extracted, and spectral profiles between 230 and 300 nm were determined.

The effect of the lead inhibitors (**3a** and **3b)** was also studied on ergosterol biosynthesis in *Candida* cells to support that azole drugs interfere with biosynthesis of cell membranes [34]. The ergosterol content of exposed cells was estimated in *C*. *albicans* ATCC 90028 and *C*. *albicans* D15.9 (FLC-resistant) strains by a previously reported method [29]. FLC was used as a positive control. The results showed a dose dependent decrease in ergosterol content when cells were grown in varying concentrations of test compounds. The mean decrease in total ergosterol content for *C*. *albicans* ATCC 90028 was found to be 48% at MIC/4, and 100% above MIC/2 of compound **3a**. The mean decrease in total ergosterol content for *C*. *albicans* D15.9 (FLC-resistant) was found to be 30% at MIC/4, and 100% above MIC/2 of compound **3a**. Similarly, the mean decrease in total ergosterol content for standard *C*. *albicans* ATCC 90028 was found to be 33% at MIC/4, and 100% above MIC/2 of **3b**. However, the decrease in total ergosterol content for *C*. *albicans* D15.9 (FLC-resistant) was found to be 23% at MIC/4, 98% at MIC/2 and 100% at MIC of compound **3b**. In the presence of 40 μg/mL of FLC, the decrease in ergosterol content was 100% and 96% respectively for *C*. *albicans* ATCC 90028 and *C*. *albicans* D15.9 (FLC-resistant). Our results suggest that the compounds **3a** and **3b** inhibit the total ergosterol content significantly for all the *Candida* strains tested here (Fig 7 and S6 Fig).

**Fig 8:**
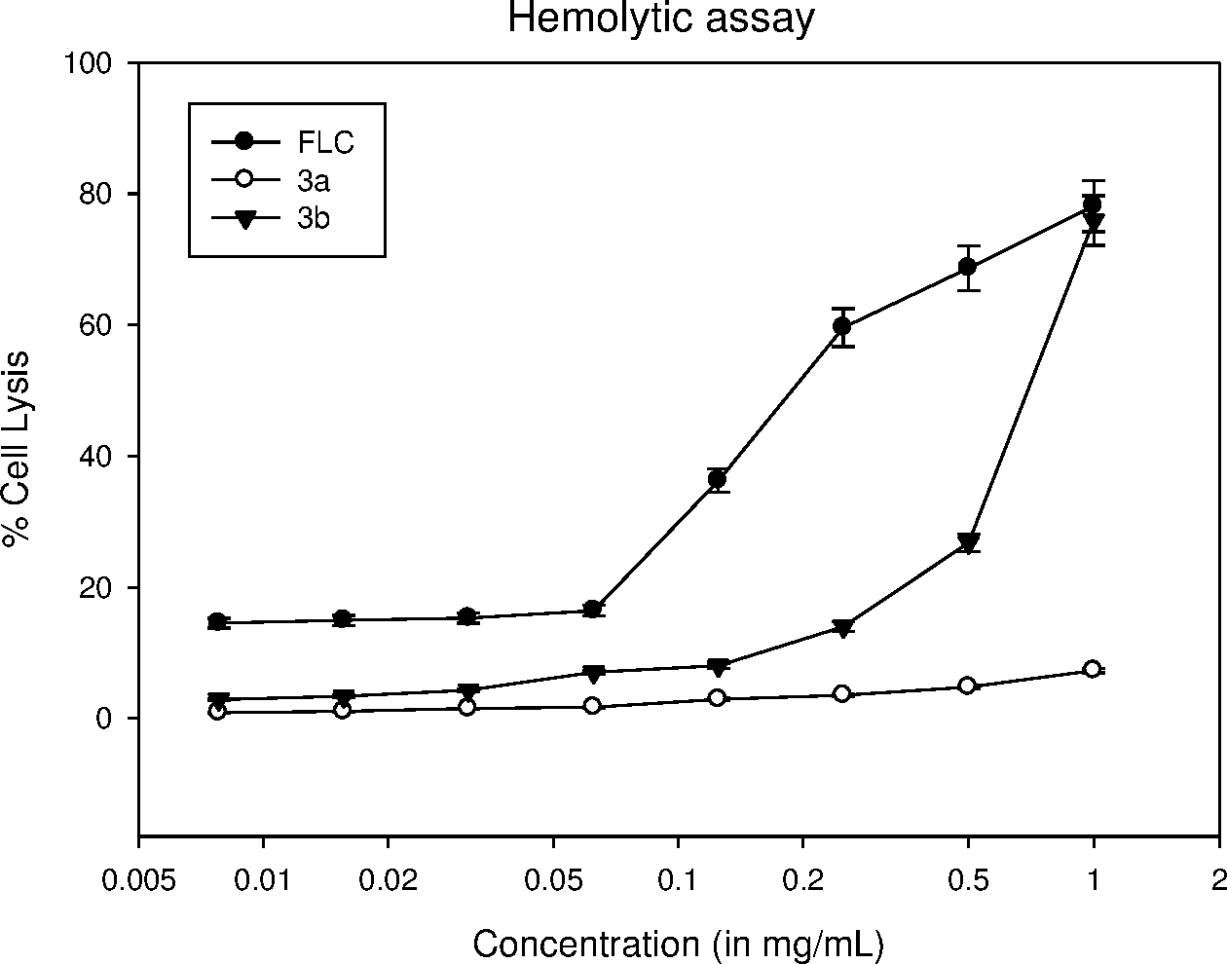
Hemolytic activity of compounds 3a, 3b and FLC. The hRBCs were collected, resuspended in PBS and treated with various concentrations of test compounds. Results show that the standard drug FLC exhibits greater cell lysis in comparison to compounds the test compounds

The cytotoxic effect of lead compounds (**3a** and **3b)** was determined on human red blood cells (hRBCs) by hemolytic assay and compared with standard drug FLC for reference. At 500 μg/mL, compounds **3a** and **3b** showed 4.76 and 26.77% hemolysis of hRBCs while at the same concentration, standard drug FLC showed 68.63% hemolysis (Fig 8). Both the test compounds showed <5% hemolysis at their respective MIC values against standard *Candida* strain. Thus the results strongly suggest that these compounds have negligible toxic effect on hRBCs. To examine the effect of triazole derivatives (**3a** and **3b)** on cell proliferation, they were screened for cytotoxicity against monkey kidney fibroblasts (VERO). A sub-confluent population of VERO cells was treated with increasing concentration of each compound and the number of viable cells was measured after 48 h by MTT cell viability assay. Doxorubicin was used as control which showed IC_50_ of >5 μg/mL. Neither compound was found cytotoxic upto 10 μg/mL.

## Discussion

*C*. *albicans* is an opportunistic fungus and is responsible for the most common fungal infection called candidiasis. These infections are continuously increasing all around the world with high mortality rate due to the limitations of current antifungal therapies. Thus, constant researches are required to generate new options for treatment of serious fungal infections with better armamentarium, low toxicity, and target specificity. Among the currently used antifungal agents, triazoles (e.g. fluconazole, itraconazole, posaconazole) are known for better therapeutic properties [35]. On the other hand, several reports including our lab have suggested towards the role of natural molecules as potential inhibitors of *Candida* spp [16, 28]. Recently we synthesized a series of 1,2,3-triazoles from naturally occurring bioactive molecules and found compound **3a** as a promising inhibitor of *Candida* spp [20]. Compound **3a** was synthesized from 8-hydroxy quinoline and we speculated that the quinoline, ring especially *N*-atom, is responsible for its enhanced anti-*Candida* activity. In this work, we synthesized and evaluated anti-*Candida* activities of two other structural analogues (**3b** and **3c**) of compound **3a** in order to determine the role of quinoline ring. We further performed various experiments to determine their possible mode of action in *C*. *albicans*. We obtained low IC_50_ values with compounds **3a** and **3b** against *Candida* spp from preliminary screening. Moreover, *in vitro* studies have shown that compounds **3a** and **3b** significantly inhibit FLC- susceptible as well as resistant clinical isolates of *C*. *albicans*. These results encouraged us to explore their possible effect on some important virulence attributes of *C*. *albicans*. We performed disk diffusion assay, growth curve studies and time kill assay to determine the fungicidal or fungistatic nature of compounds **3a** and **3b**. We demonstrated that at high concentrations compound **3a** exhibits fungicidal activity whereas compound **3b** showed fungistatic behaviour. It was noted that the performance of compound **3a** was better than the standard drug FLC. Virulence attributes in pathogens can be used as alternative targets for drug development. Secretion of proteinases and phospholipases are important virulence factors in *C*. *albicans* which help in the invasion and damage of host tissues [36]. The significant inhibition in proteinase and phospholipase secretion by triazole-amino acid hybrids has been previously reported by our group [30], which motivated us to further demonstrate the effect of compounds **3a** and **3b** on these enzymes. Secretion of proteinases and phospholipases was inhibited in the presence of compounds **3a** and **3b** but it was not enough to conclude that these proteins maybe be used as potential antifungal targets. Compound **3a** inhibited approximately two fold secretion of proteinases in FLC- resistant *C*. *albicans* and no phospholipase activity was observed in *C*. *albicans* ATCC 90028 after treatment with compound **3b**. H^+^ ATPase, a member of P-type transport ATPase family, has been reported as a potential antifungal target [37, 38]. This protein is essentially involved in the physiological functions of *Candida* spp such as maintenance of electrochemical gradient across cell membrane, nutrient uptake, regulation of intracellular pH and cell growth. H^+^ ATPase of fungal origin is not similar to the human protein and might be a useful antifungal target [17]. Some previous studies have reported that FLC inhibits H^+^ ATPase activity *in C*. *albicans* [16, 39]. Therefore, we explored the action of the test compounds on the activity of plasma membrane H^+^ ATPase. We observed good to moderate inhibition in the H^+^ ATPase activity in absence and presence of glucose when treated with 50 and 100 μg/mL of the test compounds. Moreover, we also determined the internal pH of *C*. *albicans* in presence of compounds **3a** and **3b** and the results also supported the partial inhibition of H^+^ ATPase activity. The main focus of this study was to develop some new compounds with an azole moiety. It is well known that azoles have a common mode of action as they inhibit ergosterol biosynthesis by blocking cytochrome P450-dependent enzyme: lanosterol 14 α-demethylase. Ergosterol is the main sterol constituent of fungal membranes [40]. Thus, we examined the effect of test compounds on cell membranes and ergosterol biosynthesis by transmission electron microscopy and sterol profiling, respectively. We observed alterations in the morphology of treated *C*. *albicans* cells especially in cell membrane and cell organelles in comparison to the normal cells. This indicates that the test compounds are involved in the disintegration of the fungal cell membrane. The ergosterol content was negligible after treatment with the test compounds at MIC. Additionally, cell proliferation assay and haemolytic assay showed that both compounds **3a** and **3b** were significantly less cytotoxic. Thus, overall findings of this study indicate that compounds **3a** and **3b** have good anti-*Candida* activity and the mode of action is similar to FLC. The compounds do inhibit other virulence attributes in *C*. *albicans* but the main antifungal property may be due to the inhibition of ergosterol biosynthesis. Further, pharmacodynamic studies and *in vivo* efficacy of these compounds are required to establish these compounds as future antifungal agents.

## Conclusion

The structural analogues of a previously determined anti-*Candida* compound **3a** were synthesized. Based on the preliminary anti-*Candida* results, **3a** and **3b** were selected as lead compounds and further explored for their antifungal potential against *C*. *albicans*. Both compounds showed good anti-*Candida* activity for all the strains used. This study showed that both compounds **3a** and **3b** have significant inhibitory effect on the growth of FLC- sensitive as well as FLC- resistant *C*. *albicans* strains. Growth curve and time kill curve studies indicated that compound **3a** has fungicidal nature while compound **3b** was fungistatic. The secretion of extracellular hydrolytic enzymes (proteinases and phospholipases) was affected in the presence of both the test compounds. An altered cell membrane, reduced plasma membrane H^+^ ATPase activity and significant inhibition in ergosterol biosynthesis in the presence of these compounds suggest that plasma membrane of the pathogenic fungi is an important antifungal target for the newly synthesized triazoles. Hence, we conclude that compounds **3a** and its analogue **3b** are potential anti-*Candida* compounds with mode of action similar to FLC.

## Supporting information

S1 Fig ^1^H NMR spectra of compound 3b. (JPG)

S2 Fig ^13^C NMR spectra of compound 3b. (JPG)

S3 Fig Disk Diffusion Assay. Disk diffusion assay of standard, FLC-susceptible and FLC-resistant *C*. *albicans* showing zone of inhibition in the presence of different concentration of test compounds 3a and 3b.
(TIF)

S4 Fig H^+^ efflux activity. Inhibition of the rate of H^+^ efflux by *C*. *albicans* ATCC 90028; FLC-susceptible and FLC-resistant isolate of *Candida* in presence and absence of glucose in presence of compounds 3a and 3b, a) without glucose; b) with glucose.Error bars represents Mean±S.D. from three independent recordings.
(TIF)

S5 Fig Ergosterol Inhibition Assay. Percentage inhibition of ergosterol in a) standard strain; and b) resistant strain showing by bar graph in presence of compounds 3a and 3b. Error bars represents mean±S.D. from three independent recordings.
(TIF)

S6 Fig (a-c). Phase contrast microscopy. Phase contrast microscopy was performed to determine the effect of lead inhibitor on the morphology of *C*. *albicans*. Mid-log phase cells were harvested, standardized (A600 ≈ 0.1) and treated with the MIC concentration of lead inhibitors 3a and 3b for 6 h. After treatment period, cells were washed thrice with phosphate buffer solution to remove residual medium. 10 μL of cell suspension was put over a clean glass slide and observed under phase contrast microscope (phase plate 3, Nikon Eclipse 80i). The morphological differences between a) un-treated cells of *C*. *albicans* and b-c) treated with compound 3a and 3b, respectively were observed.
(TIF)

## Abbreviations

FLC: Fluconazole
ATCC: American Type Culture Collection
MIC: Minimum Inhibitory Concentration
IC_50_: Concentration required for 50% inhibition
CDC: Center for Disease Control and Prevention
CDCl_3_: Chloroform
DCM: Dichloromethane
DMF: Dimethylformamide
TLC: Thin Layer Chromatography
DMSO: Dimethyl sulfoxaide
SD: Sabouraud Dextrose
YEPD: Yeast Extract Peptone Dextrose
h: hour/s
TEM: Transmission Electron Microscopy
DHE: Dehydroergosterol
ZOI: Zone of inhibition
