# Supplementary figures and images for: Effect of quinoline based 1,2,3-triazole and its structural analogues on growth and virulence attributes of Candida albicans
Source: PLoS One. 2017 Apr 21;12(4):e0175710. doi: 10.1371/journal.pone.0175710 (PMC5400251; doi:10.1371/journal.pone.0175710)

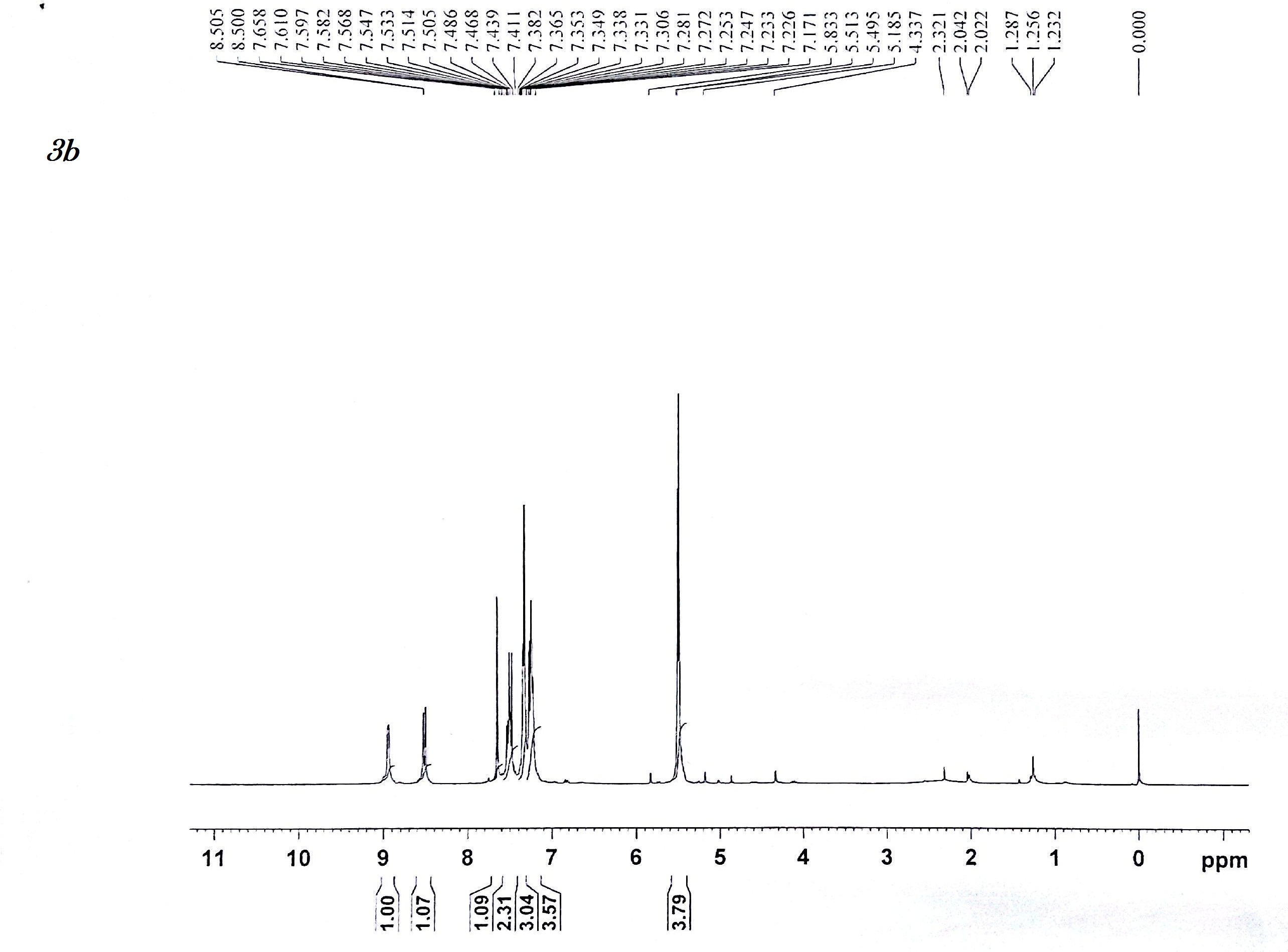

Supplement: S1 Fig — (JPG) [file pone.0175710.s001.jpg]

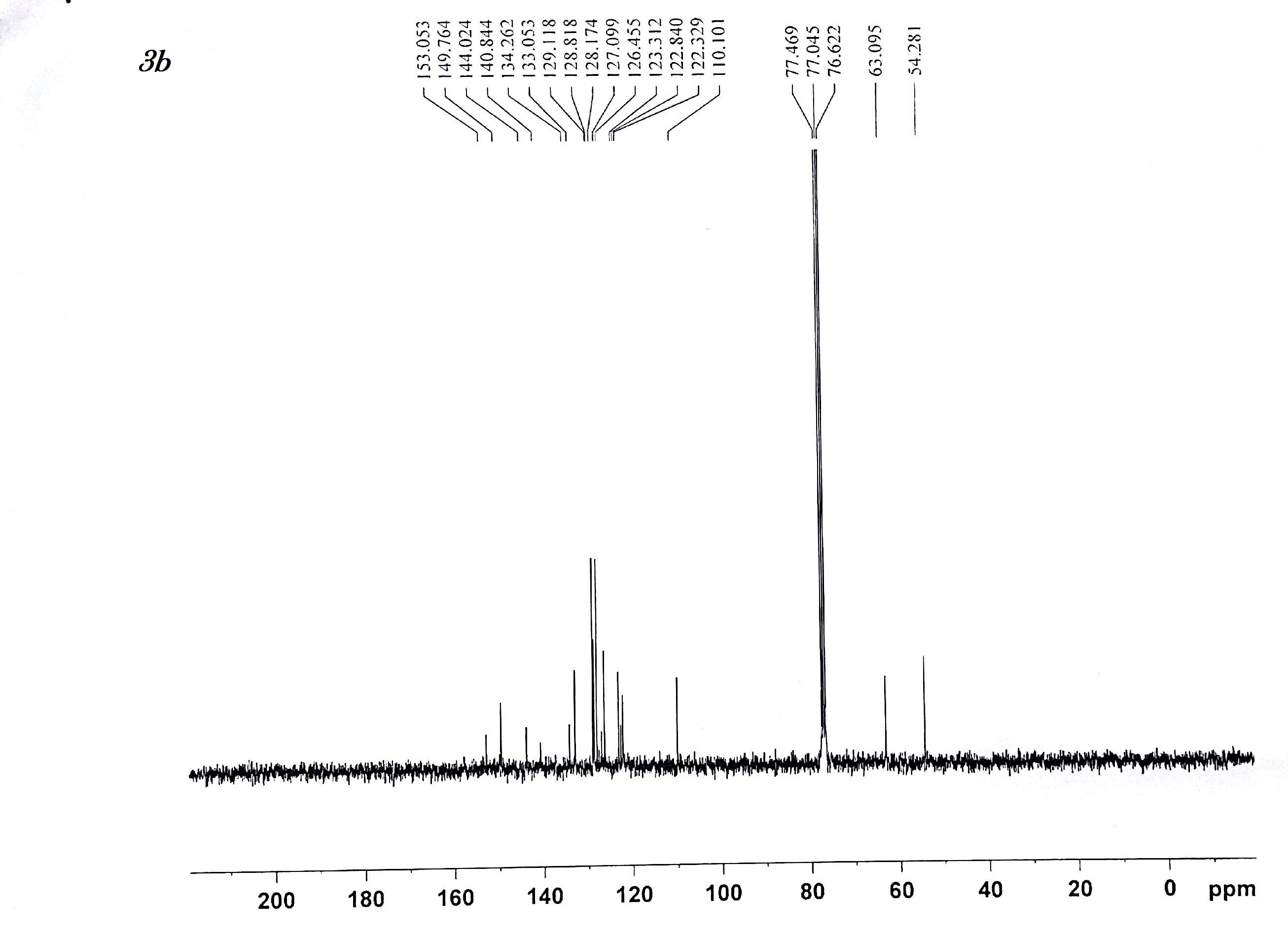

Supplement: S2 Fig — (JPG) [file pone.0175710.s002.jpg]

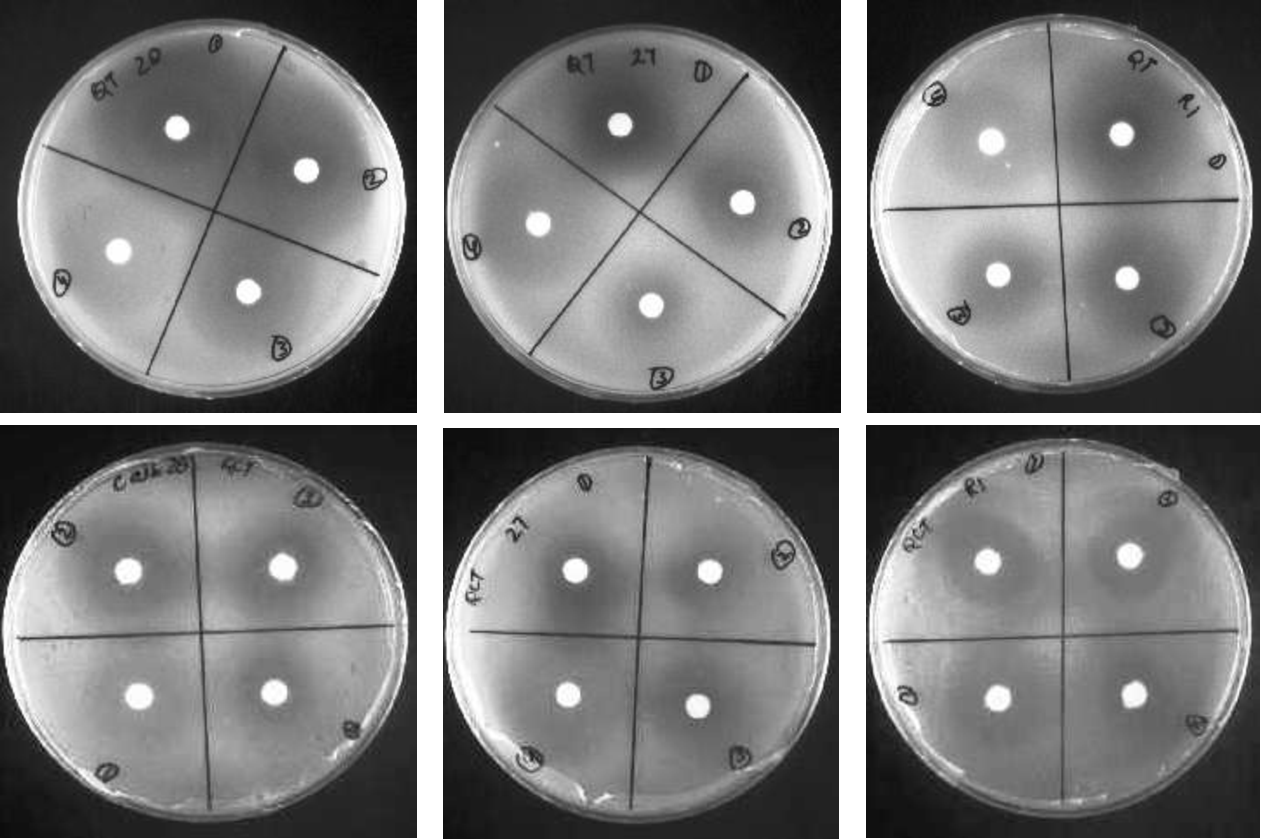

Supplement: S3 Fig — Disk diffusion assay of standard, FLC-susceptible and FLC-resistant C. albicans showing zone of inhibition in the presence of different concentration of test compounds 3a and 3b. (TIF) [file pone.0175710.s003.tif]

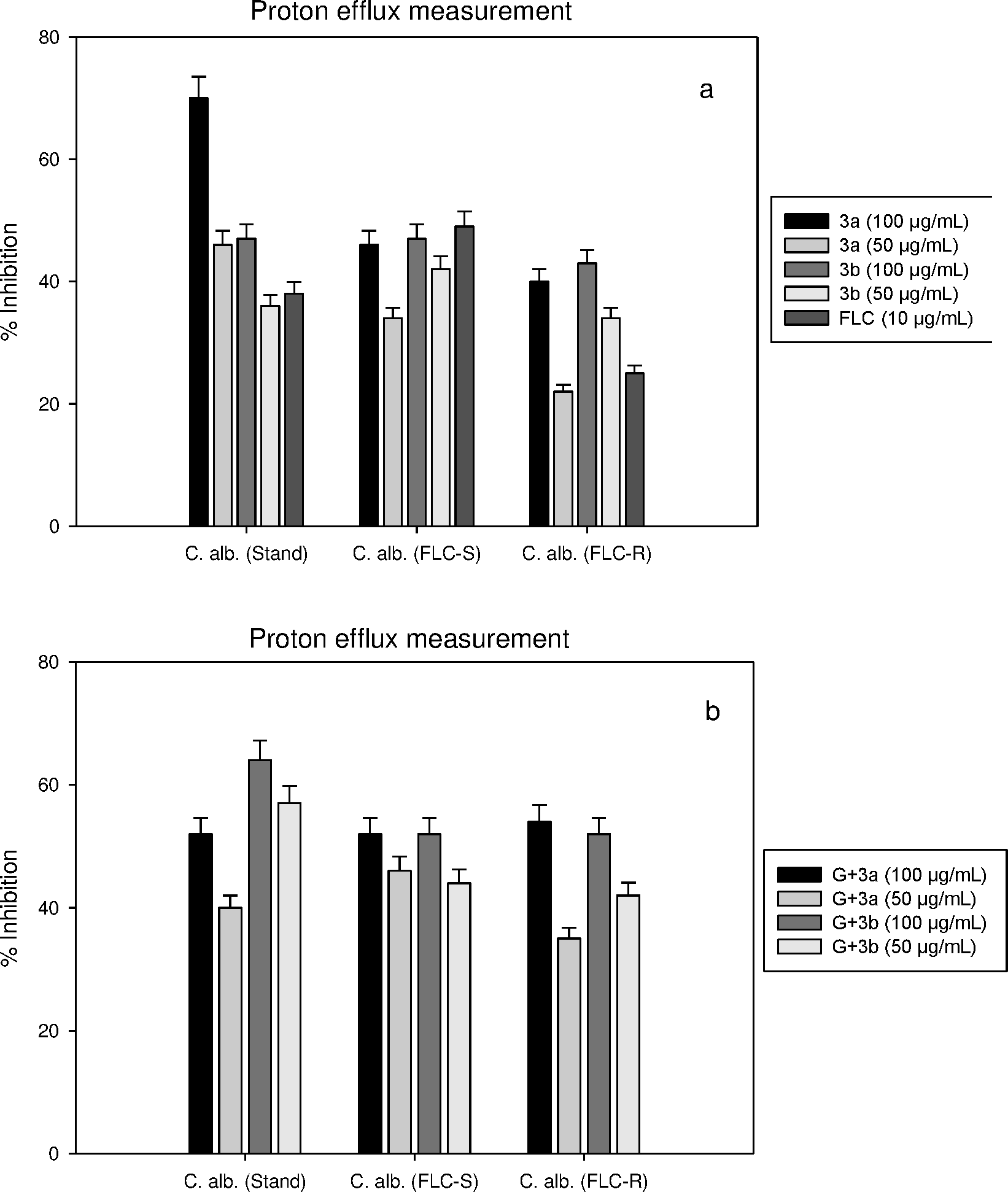

Supplement: S4 Fig — Inhibition of the rate of H+ efflux by C. albicans ATCC 90028; FLC-susceptible and FLC-resistant isolate of Candida in presence and absence of glucose in presence of compounds 3a and 3b, a) without glucose; b) with glucose.Error bars represents Mean±S.D. from three independent recordings. (TIF) [file pone.0175710.s004.tif]

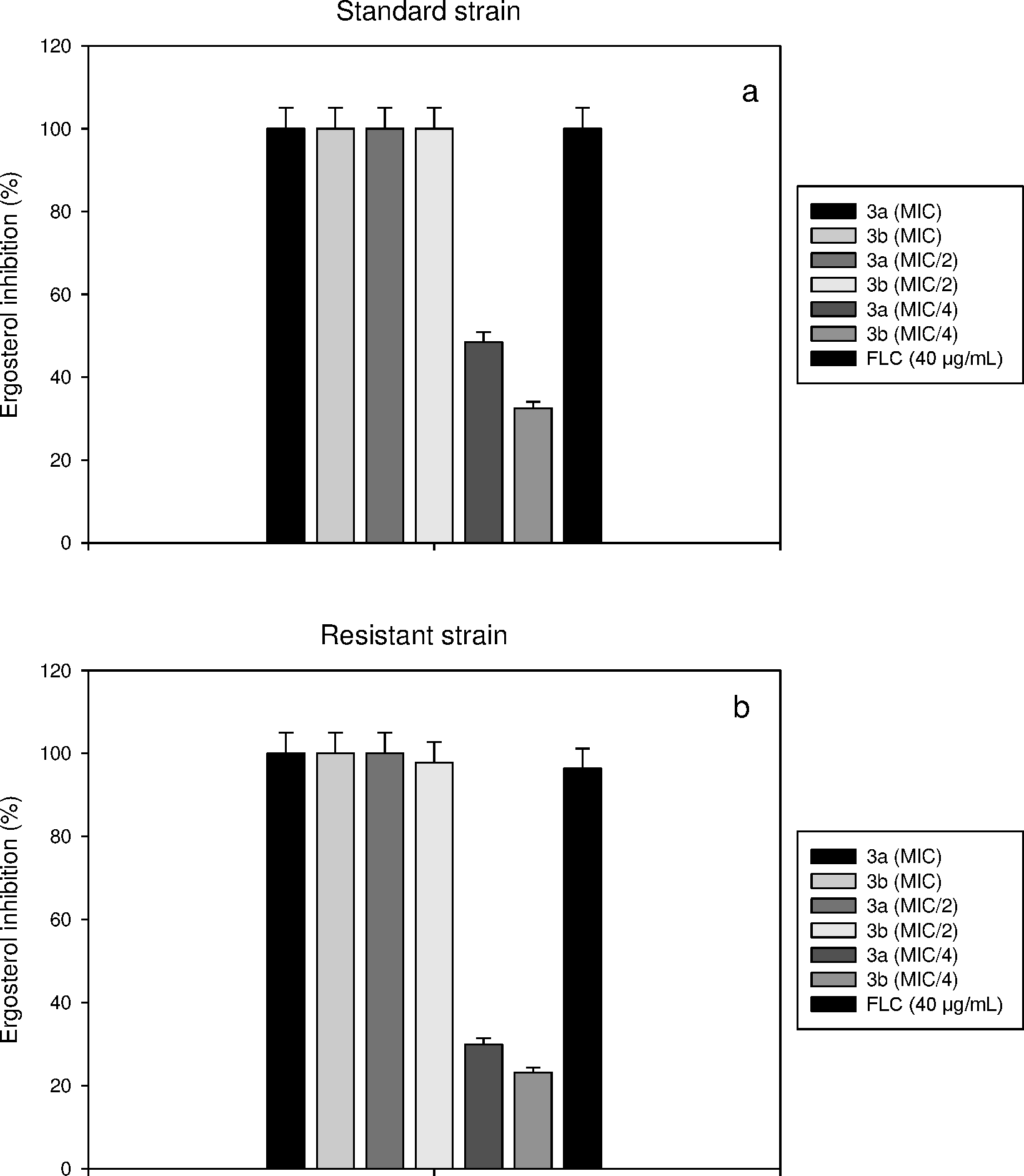

Supplement: S5 Fig — Percentage inhibition of ergosterol in a) standard strain; and b) resistant strain showing by bar graph in presence of compounds 3a and 3b. Error bars represents mean±S.D. from three independent recordings. (TIF) [file pone.0175710.s005.tif]

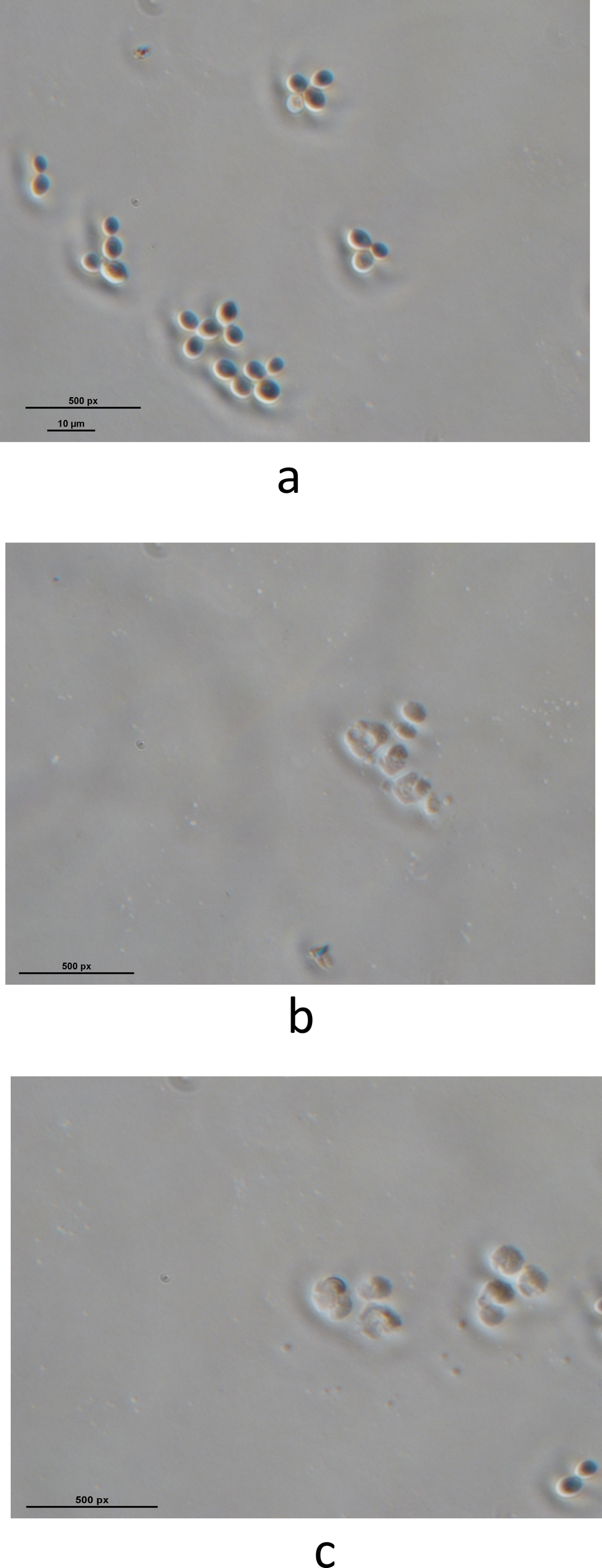

Supplement: S6 Fig — (a-c). Phase contrast microscopy. Phase contrast microscopy was performed to determine the effect of lead inhibitor on the morphology of C. albicans. Mid-log phase cells were harvested, standardized (A600 ≈ 0.1) and treated with the MIC concentration of lead inhibitors 3a and 3b for 6 h. After treatment period, cells were washed thrice with phosphate buffer solution to remove residual medium. 10 μL of cell suspension was put over a clean glass slide and observed under phase contrast microscope (phase plate 3, Nikon Eclipse 80i). The morphological differences between a) un-treated cells of C. albicans and b-c) treated with compound 3a and 3b, respectively were observed. (TIF) [file pone.0175710.s006.tif]
